# Inhibition of Cancer Cell Proliferation and Bacterial Growth by Silver(I) Complexes Bearing a CH_3_-Substituted Thiadiazole-Based Thioamide

**DOI:** 10.3390/molecules28010336

**Published:** 2023-01-01

**Authors:** Despoina Varna, Elena Geromichalou, Georgia Karlioti, Rigini Papi, Panagiotis Dalezis, Antonios G. Hatzidimitriou, George Psomas, Theodora Choli-Papadopoulou, Dimitrios T. Trafalis, Panagiotis A. Angaridis

**Affiliations:** 1 Laboratory of Inorganic Chemistry, Department of Chemistry, Aristotle University of Thessaloniki, 54124 Thessaloniki, Greece; 2 Laboratory of Pharmacology, Medical School, National and Kapodistrian University of Athens, 75 Mikras Asias Street, 11527 Athens, Greece; 3 Laboratory of Biochemistry, Department of Chemistry, Aristotle University of Thessaloniki, 54124 Thessaloniki, Greece

**Keywords:** silver(I) complexes, phosphines, heterocyclic thioamides, antibacterial and anti-cancer properties, radical scavenging, interaction with biomacromolecules, molecular docking, FGFR1

## Abstract

Ag(I) coordination compounds have recently attracted much attention as antiproliferative and antibacterial agents against a wide range of cancer cell lines and pathogens. The bioactivity potential of these complexes depends on their structural characteristics and the nature of their ligands. Herein, we present a series of four Ag(I) coordination compounds bearing as ligands the CH_3_-substituted thiadiazole-based thioamide 5-methyl-1,3,4-thiadiazole-2-thiol (mtdztH) and phosphines, i.e., [AgCl(mtdztH)(PPh_3_)_2_] (**1**), [Ag(mtdzt)(PPh_3_)_3_] (**2**), [AgCl(mtdztH)(xantphos)] (**3**), and [AgmtdztH)(dppe)(NO_3_)]_n_ (**4**), where xantphos = 4,5-bis(diphenylphosphino)-9,9-dimethylxanthene and dppe = 1,2-bis(diphenylphosphino)ethane, and the assessment of their in vitro antibacterial and anti-cancer efficiency. Among them, diphosphine-containing compounds **3** and **4** were found to exhibit broad-spectrum antibacterial activity characteristics against both Gram-(+) and Gram-(–) bacterial strains, showing high in vitro bioactivity with IC_50_ values as low as 4.6 μΜ. In vitro cytotoxicity studies against human ovarian, pancreatic, lung, and prostate cancer cell lines revealed the strong cytotoxic potential of **2** and **4**, with IC_50_ values in the range of 3.1–24.0 μΜ, while **3** and **4** maintained the normal fibroblast cells’ viability at relatively higher levels. Assessment of these results, in combination with those obtained for analogous Ag(I) complexes bearing similar heterocyclic thioamides, suggest the pivotal role of the substituent groups of the thioamide heterocyclic ring in the antibacterial and anti-cancer efficacy of the respective Ag(I) complexes. Compounds **1**–**4** exhibited moderate in vitro antioxidant capacity for free radicals scavenging, as well as reasonably strong ability to interact with calf-thymus DNA, suggesting the likely implication of these properties in their bioactivity mechanisms. Complementary insights into the possible mechanism of their anti-cancer activity were provided by molecular docking calculations, exploring their ability to bind to the overexpressed fibroblast growth factor receptor 1 (FGFR1), affecting cancer cells’ functionalities.

## 1. Introduction

Cancer is a leading cause of millions of deaths worldwide and, according to the International Agency for Research on Cancer, a rapid increase in new cancer cases is predicted by 2030 [1]. Despite the substantial progress made in medicine, the understanding and treatment of particular types of cancer remain a great challenge and, therefore, the development of novel anti-cancer drugs becomes an urgent need. Shortcomings of cisplatin cancer treatment (mainly toxicity and tumor drug resistance) motivated research towards the discovery of new chemotherapeutic agents based on other transition metals, such as ruthenium, iridium, and gold. Among them, Ag(I) complexes recently attracted much attention as promising antiproliferative agents [2,3,4,5]. However, available data on their effective bioactivity against different human cancer cells are still limited.

A family of Ag(I) complexes exhibiting high inhibitory potential for cancer cells proliferation involves those with heterocyclic thioamide ligands. Heterocyclic thioamides are able to adopt different coordination modes (depending on their protonation state), e.g., monodentate (κ*S* or κ*Ν*), bidentate chelating (κ*S,Ν*) or bidentate bridging (μ-*S,N*), allowing them to act as multifunctional ligands and lead to the formation of metal complexes with diverse structural characteristics (e.g., total charge, nuclearity, metal coordination number, and geometry) [6]. Moreover, the structural resemblance of these organic compounds with basic building blocks of biological entities allows for the development of strong intermolecular interactions with various biomacromolecules, providing them with interesting biological properties and bioactivity [7]. Five-membered heterocyclic ring thioamides, and, in particular, those containing the 1,3,4-thiadiazole ring, have been reported to possess significant antiproliferative activity against different human cancer cells, a fact that was attributed, to a large extent, to the high electron-donating ability of their N atoms and their ability to interact through H-bonding with various biomolecular targets involved in cancer cell proliferation and metastasis. Moreover, 1,3,4-thiadiazole derivatives, which are bioisosteres of pyrimidine, were found to be able to interfere with DNA replication, regulate angiogenesis, or control the cell cycle in cancer cells while some of them have been used in clinical phases I-II, showing encouraging perspectives as alternatives in cancer treatment [8,9,10,11]. 

Among the Ag(I) complexes bearing small-ring heterocyclic thioamides, those containing phosphine co-ligands appear to be an intriguing case, owing to the fact that they combine the unique bioactivity properties of thioamides with the beneficial properties of phosphine ligands. In particular, phosphine ligands are known for their ability to improve the kinetic stability of their Ag(I) complexes in biological environments and provide a balanced lipophilic/hydrophilic character that is necessary for efficient biological activity of the complexes. Indeed, a number of thioamide/phosphine Ag(I) complexes have been reported to exhibit significant in vitro bioactivity against a range of different cancer cell lines and bacterial strains. For example, the anti-cancer activity potential of [AgCl(CMBZT)(TPTP)_2_], where CMBZT = 5-chloro-2-mercaptobenzothiazole and TPTP = tris(p-tolyl)phosphine, against leiomyosarcoma cancer cells was found to be higher than that of cisplatin [12] The structurally analogous complex [AgI(PPh_3_)_2_(MBZT)], where MBZT = 2-mercapto-benzothiazole, was tested for its cytotoxic efficacy upon or without UVB irradiation against a series of cancer cells, and found to display increased (higher than cisplatin) cytotoxicity upon irradiation [13]. In a related study, Ag(I) complexes bearing imidazolidine-2-thione or benzimidazoline-2-thione ligands were reported to exhibit enhanced biological activity, compared to AgNO_3_, against a series of different cancer cells, a result that was correlated with their balanced hydrophilic–lipophilic nature offered by the phosphine co-ligands [14]. Concerning the antibacterial potential of this family of Ag(I) complexes, it has been reported that [Ag(Imt)_2_(PPh_3_)](NO_3_), where Imt = imidazolidine-2-thione, displays significant activity against the Gram-(–) *E. coli* and *P. aeruginosa* bacterial strains [15]. Mononuclear and binuclear Ag(I) complexes bearing an imidazolidine-2-thione ligand, such as 1-methyl-imidazolidine-2-thione, 1-ethyl-imidazolidine-2-thione, 1-n-propyl-imidazolidine-2-thione, 1-n-butyl-imidazolidine-2-thione, and 1-phenyl-imidazolidine-2-thione, and PPh_3_ co-ligands were also found to be effective against methicillin-resistant *S. aureus*, *K. pneumoniae,* and *S. typhimurium* bacteria [16]. Finally, in another study, cationic mixed-ligand thioamide/phosphine Ag(I) complexes [Ag_2_(Cy_3_P)_2_(tzdSH)_2_](PF_6_)_2_ and [Ag(Cy_3_P)(tzdSH)_2_](NO_3_), where tzdSH = thiazolidine-2-thione and Cy_3_P = tricyclohexylphosphine, were reported to display moderate activity against the Gram-(–) *P. vulgaris, P. aeruginosa, E. coli,* and *K. pneumoniae* bacteria [17]. 

Studies carried out in our laboratory on Ag(I) complexes bearing the five-membered heterocyclic ring thioamides 4-phenyl-imidazole-2-thione (phimtH) and 2,2,5,5-tetramethyl-imidazolidine-4-thione (tmimdtH) and phosphines also revealed their moderate-to-high in vitro cytotoxic and antibacterial potential against a variety of human cancer cell lines and bacterial strains, respectively [18]. More recently, we reported a series of heteroleptic Ag(I) complexes bearing a five-membered heterocyclic ring thioamide with group substituents of high electronegativity, i.e., 5-amino-1,3,4-thiadiazole-2(3H)-thione (atdztH) and 4-methyl-5-(trifluoromethyl)-1,2,4-triazol-3-thiol (mtftH), and the results of their in vitro anti-cancer and antibacterial activity assessment [19,20,21]. Noticeably, high antiproliferative activity against various human cancer cells, together with some degree of selectivity against normal cells, and moderate-to-high antibacterial activity against various Gram-(+) and Gram-(‒) bacteria was observed for the complexes bearing the NH_2_-substituted thioamide, while the CF_3_-substitution of the thioamide proved to be detrimental for the observed bioactivity. Undoubtedly, the introduction of group substituents of different electronegativity and lipophilicity on the heterocyclic ring of the thioamide has a determining effect on the bioactivity of their corresponding complexes.

**Scheme 1:**
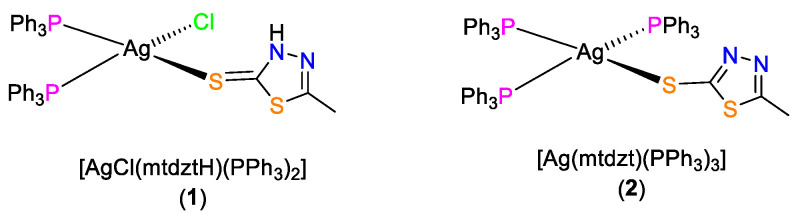
Molecular structures of **1** and **2**.

These findings provided us with promising perspectives and a good initiative for further study of the bioactivity potential of Ag(I) complexes of this family. A particularly interesting problem is the investigation of the impact of variation in group substituents of the five-membered heterocyclic ring of the thioamide ligands on the biological potency of the respective Ag(I) complexes. To this end, herein, we report a series of Ag(I) complexes bearing the CH_3_-substituted thiadiazole-based thioamide 1,3,4-thiadiazole-2(3H)-thione (mtdztH) and phosphines, and the in vitro study of their potential for growth inhibition of a variety of human cancer cell lines and bacterial strains. In particular, the mononuclear complex [AgCl(mtdztH)(xantphos)] and the polymeric compound [Ag(mtdztH)(dppe)(NO_3_)]_n_, where xantphos = 4,5-bis(diphenylphosphino)-9,9-dimethylxanthene and dppe = 1,2-bis(diphenylphosphino)ethane, were synthesized. The Ag(I) complexes bearing the same thioamide [AgCl(mtdztH)(PPh_3_)_2_] (**1**) and [Ag(mtdzt)(PPh_3_)_3_] (**2**) (Scheme 1), which we reported recently [20], were also synthesized for comparison reasons. The in vitro antibacterial efficiency of the four complexes was evaluated against the Gram-(+) (*S. aureus, B. cereus, B. subtilis*) and Gram-(–) (*E. coli*) bacterial strains, while their in vitro cytotoxicity was explored against human ovarian (SKOV-3), lung (DMS-114), pancreas (Hup-T3), and prostate (PC3) cancer cells, and the fetal lung fibroblast (MRC-5) cells. The varying individual structural characteristics of these Ag(I) compounds, e.g., nuclearity, metal coordination number, and geometry, protonation state of the thioamide, phosphine denticity and coordination mode, are expected to influence their pharmacokinetic properties, regarding their stability in biological media and their efficient cellular uptake, and, therefore, their bioactivity. Therefore, the herein-presented bioactivity study provides the opportunity to infer potential structure/bioactivity correlations: (a) among the studied Ag(I) compounds and (b) among the members of the broader family of Ag(I) compounds bearing a five-membered heterocyclic ring thioamide with different ring-substituents, i.e., CH_3_- vs. NH_2_- and CF_3_ groups. Finally, in vitro studies of the interaction of the Ag(I) compounds with calf thymus (CT) DNA and serum albumins were employed to assess their potential to bind to bio-macromolecular targets and drug carriers, whereas molecular docking calculations on the overexpressed Fibroblast Growth Factor Receptor 1 (FGFR1) in the aforementioned cancer cells provided useful information for the likely implication of these properties in their bioactivity mechanisms.

## 2. Results and Discussion

### 2.1. Synthesis and Characterization

Compounds **1**–**4** were synthesized following a general one-pot/two-step experimental procedure which involved an initial reaction of a Ag(I) salt with a phosphine in an appropriate solvent, followed by the addition of the thioamide, either in its neutral or deprotonated form. In particular, mononuclear complexes **1** and **3**, having the general formula [AgCl(mtdztH)(PP)], where PP = 2PPh_3_ (**1**) and PP = xantphos (**3**), were obtained upon the treatment of AgCl with 2 equiv of PPh_3_ or 1 equiv of xantphos, respectively, followed by the addition of 1 equiv of the neutral heterocyclic thioamide mtdztH in a CH_3_CN/CH_3_OH mixture under reflux. The reaction of equimolar amounts of AgBF_4_ and K^+^mtdzt^–^ in the presence of a two-fold excess of PPh_3_ under mild conditions in a CH_3_CN/CH_3_OH mixture afforded the mononuclear complex [Ag(mtdtzt)(PPh_3_)_3_] (**2**). Finally, the addition of 1 equiv of mtdtztH to an equimolar mixture of AgNO_3_ and dppe in CH_2_Cl_2_, at room temperature, resulted in the polymeric compound [Ag(mtdztH)(dppe)(NO_3_)]_n_ (**4**). All compounds were isolated in the form of white microcrystalline solids in good yields (~50%) after filtration and subsequent crystallization from their respective filtrates. They are stable in air, both in the solid state and in solution in common organic solvents, for long periods of time.

**Figure 1:**
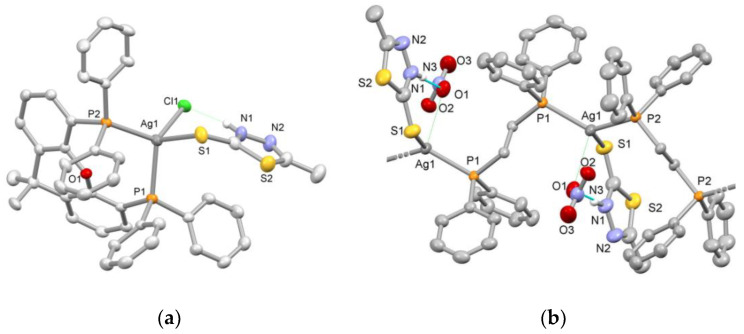
Views of the X-ray crystal structures of (**a**) [AgCl(mtdztH)(xantphos)] (**3**) and (**b**) part of the polymeric chain of [Ag(mtdztH)(dppe)(NO_3_)]_n_ (**4**). Displacement ellipsoids are drawn at the 35% probability level. Hydrogen atoms, except those of the NH groups of the thioamides, are omitted for clarity. Intramolecular hydrogen-bonding interactions are shown with dotted lines.

Single-crystal X-ray diffraction analysis revealed the solid-state structures of the four compounds. The crystal structures of **1** and **2** were presented in our previous work [20] and, therefore, they are not discussed herein. The details of crystal data and structure refinement parameters for **3** and **4** are given in Table S1. Views of their crystal structures are illustrated in Figure 1, while selected bond lengths and bond angles are provided in Table 1 (also in Figures S1 and S2 and Tables S2 and S3, respectively).

Compound **3** comprises a central metal atom surrounded by the P atoms of a chelating xantphos ligand, the exocyclic S atom of a terminally coordinated neutral mtdztH, and a Cl atom (Figure 1a). The coordination sphere of the donor atoms around the metal center exhibits slight deviations from the ideal tetrahedral geometry, with bond angles ranging between 99.98(14) and 121.74(3)°. In particular, the geometry index τ_4_ for the metal center was calculated using the equation τ_4_ = [360 − (α + β)]/141 proposed by Houser and coworkers [22], where α and β are the two largest bond angles around the metal center. τ_4_ values range between 0.0 for the perfect square planar geometry and 1 for the ideal tetrahedral geometry. The calculated τ_4_ value for **3** was found to be [360 − (121.74 + 113.00)]/141 = 0.88 which is close to the ideal tetrahedral geometry, as expected for a four-coordinate d^10^ metal ion without ligand geometrical constraints. The compound displays two unequal Ag‒P bonds with lengths of 2.513(1) Å and 2.471(1) Å. The Ag‒S bond length was found to be at 2.699(2) Å which is similar to the corresponding one found in other complexes of the general type [AgCl(thioamide)(PPh_3_)_2_] and [AgCl(thioamide)(P^^^P)] (P^^^P = diphosphine), reported in our previous work [21]. Finally, **3** is stabilized by intramolecular hydrogen bonding between the NH group of the thioamide ligand and the coordinated halogen atom, as revealed by the relative orientation of mtdztH in reference to the Cl atom and the short Cl···N interatomic distance of 3.089 Å.

Compound **4** is a one-dimensional coordination polymer with the formula [Ag(mtdztH)(dppe)(NO_3_)]_n_. As shown in Figure 1b, it consists of Ag atoms which are interconnected by bridging dppe ligands forming a one-dimensional zigzag chain. Each Ag atom completes its coordination sphere by the P atoms of two different dppe moieties and the exocyclic S atom of a thioamidato ligand. The Ag‒P bond lengths were found to be equal to 2.451(1) Å and 2.449(1) Å, respectively, while the Ag‒S bond length was 2.654(1) Å, values which fall within the usual ranges for analogous Ag(I) complexes reported in the literature [18,23]. The local symmetry around each Ag atom approximates a pyramidally distorted trigonal arrangement. Presumably, this might be related to the presence of weak interactions between the Ag atoms and the NO_3_ moieties in a direction perpendicular to the P_2_S plane, which is consistent with the weak coordinating ability of the NO_3_^–^ ions. Indeed, the Ag···ONO_2_ distances were found to be 2.769 Å, which are less than the sum of the van der Waals radii (3.20 Å) of Ag and O [24]. Other angles around the metal center range between 102.89(3)^o^ and 130.69(3)^o^. Intramolecular hydrogen-bonding interactions are also developed between an O atom of a weakly coordinating NO_3_ moiety with the NH group of the thioamide ligand. Views of the packing of **4** in the solid state, showing parallel one-dimensional zigzag chains extending along the a-axis, are given in Figure S3.

Infrared spectroscopy was utilized to verify the identity of the synthesized Ag(I) compounds as isolated as microcrystalline solids in bulk form. In general, experimental data obtained from the FTIR spectra of the compounds in the 4000–400 cm^−1^ region are in accordance with the findings of X-ray crystal structure determinations. The existence of characteristic absorption bands of the heterocyclic thioamide (or thioamidato) and phosphine ligands provide evidence for their coordination to the metal center. Moreover, particular thioamide absorption bands have high diagnostic value. In the FTIR spectrum of mtdztH in its free form, two intense bands assigned to C=N bond vibration located at 1553 cm^−1^ and 1464 cm^−1^ appeared to be shifted to 1556 cm^−1^ and 1473 cm^−1^, respectively, in the FTIR spectrum of **3**. The two bands of the free ligand appeared to be shifted to 1484 cm^−1^ and 1434 cm^−1^, respectively, in the spectrum of **4**. Furthermore, the absorption band of free mtdztH at 1270 cm^−1^ was attributed to the vibration of the thioamide group [25]. In the FTIR spectra of **1** and **3**, this band was shifted to higher frequencies, at 1278 cm^−1^ and 1273 cm^−1^, respectively, in agreement with the electron shift $H\vec{\ddot{N}-}C\vec{=S}:\to \mathrm{Ag}$ upon the coordination of mtdztH in its thioketo form through the exocyclic S atom to the metal center. In a similar manner, the absorption band at 767 cm^−1^ in the spectrum of mtdztH assigned to the C=S stretching [26], underwent a shift at 792 cm^−1^ and 693 cm^−1^ in the spectra of **3** and **4**, respectively. Since both **3** and **4** bear mtdztH in its neutral form, the presence of an absorption band at 3445 cm^−1^ can be attributed to the N‒H bond stretching vibration of the thioamide group.

Aiming to study the antibacterial and anti-cancer properties of the herein-presented Ag(I) compounds, it is important to examine their stability in solution, as their speciation in solution is an important factor for understanding their bioactivity. For this reason, the stability of the four compounds in solution was examined by ^1^H-NMR spectroscopy. ^1^H-NMR spectra of the compounds in CDCl_3_ displayed characteristic signals that correspond to Hs of their respective ligands. For example, the ^1^H-NMR spectrum of **3** showed a broad signal in the low-field region at 13.30 ppm which can be attributed to the H of the NH group of mtdztH, indicating its coordination through the exocyclic S atom. Moreover, the formation of the intramolecular hydrogen bond between the NH group of the thioamide and the highly electronegative coordinated Cl atom led to deshielding of the particular H and the appearance of its shift at 13.30 ppm. Signals of Hs of the Ph groups of xantphos appeared in the 7.37–7.07 ppm region as a set of partially resolved multiplets. Similarly, Hs of the diphosphine’s backbone gave multiple signals in the same region. A singlet observed at 2.47 ppm was attributed to the three Hs of the thioamide’s CH_3_ group, whereas Hs of the CH_3_ group of the diphosphine ligand gave a singlet in the high-field region (1.67 ppm), presumably as a result of the dynamic behavior of the xantphos backbone. In the ^1^H-NMR spectrum of compound **4**, partially resolved multiplets observed in the 7.75-7.36 ppm region can be attributed to the ortho-, para-, and meta-Hs of the Ph groups dppe. Signals attributed to Hs of the backbone of dppe and the CH_3_ group of mtdztH were located in the high-field region, at 2.52–2.51 ppm (doublet) and 2.78 ppm (singlet), respectively. Considering the polymeric solid-state structure of **4**, the observance of an ^1^H-NMR spectrum in CDCl_3_ suggests that, in solution, it dissociates into positively charged monomeric or oligomeric species. The exact nature and composition of the species responsible for the observed ^1^H-NMR spectrum is unknown, and fast equilibria between different monomeric and/or oligomeric species might also not be excluded. Recording the ^1^H-NMR spectra of **1**–**4** over a period of 5 days on a daily basis revealed no changes in resonances and multiplicities of their respective signals, suggesting the long-term stability of the compounds in their solutions in organic solvents. 

The long-term stability of the Ag(I) compounds was also examined in DMSO/PBS 1:2 *v*/*v* mixtures (PBS = phosphate-buffered saline at pH = 7.4) by UV–visible electronic spectroscopy. As shown in Figure S4, no changes in the pattern of the UV–visible absorption spectra of all complexes were observed between the initial spectra and those measured after 48 h, suggesting the retention of the structures of the compounds in an aqueous environment over this period of time. 

### 2.2. Photophysical Properties

**Figure 2:**
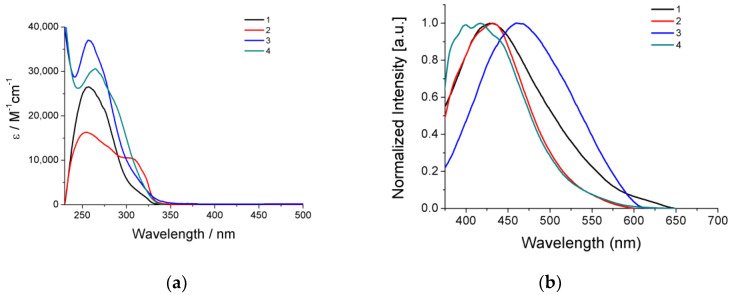
(**a**) UV–visible electronic absorption spectra of compounds **1**–**4** in CH_2_Cl_2_ solutions (~5 × 10^‒5^ M). (**b**) Emission spectra of compounds **1**–**4** in DMSO solutions (~5 × 10^‒5^ M).

UV–visible electronic absorption spectra of the Ag(I) compounds **1**–**4** in CH_2_Cl_2_ solutions are dominated by high-intensity bands in the high-energy region with wavelength absorption maxima λ_max_(abs) between 250 nm and 270 nm and their corresponding molar absorption coefficients ε ranging from 1.6 × 10^4^ to 3.7 × 10^4^ M^−1^cm^−1^ (Table 2 and Figure 2a) Regarding the absorption spectra of the respective phosphine and thioamide ligands which display absorption maxima in the same region with the complexes, the high-energy bands of the compounds could be attributed to intra-ligand *π*^∗^←*π* transitions of the aromatic groups. In addition, isostructural complexes **1** and **3**, bearing mtdztH in its neutral form, showed absorption bands at a lower intensity, at 307 and 310 nm, respectively, which can be attributed to a thioamide-originating transition.

The photoluminescence properties of the Ag(I) compounds **1**–**4** were examined in DMSO solutions. All compounds were found to be emissive showing broad emission bands in the 350–600 nm region with their maxima λ_max_(em) falling between 400 nm and 460 nm (Table 2 and Figure 2b). Complexes **1** and **3**, which are isostructural, displayed slightly different λ_max_(em) located at 428 and 432 nm, respectively. The small wavelength difference may be attributed to their different phosphine ligands. Complex **2**, bearing the thioamide in its deprotonated form, exhibited a red-shifted emission band with respect to **1**, **3**, and **4**, showing λ_max_(em) = 460 nm, a fact that gives an indication that the protonation state of the thioamide affects the emission properties of this family of complexes. In the particular case of **4**, a finely structured emission band was observed with three closely spaced maxima between 400 nm and 434 nm. Considering the similarity of the characteristics of the emission band of **4** (in terms of fine structure and wavelength emission maxima) with that of mtdztH in free form, it can be suggested that the emitting excited states of the species that exist in solution (monomers and/or oligomers) have a distinct thioamide-based character. Based on the aforementioned discussion, it may be suggested that the photoluminescence properties of the Ag(I) compounds arise from intra-ligand charge-transfer electronic transitions which involve both the thioamide (or thioamidate) and phosphine ligands. These properties allow the potential utilization of the compounds as bifunctional cell-imaging agents, provided that they also demonstrate sufficient efficacy against cancer cells.

### 2.3. In Vitro Antibacterial Activity 

**Figure 3:**
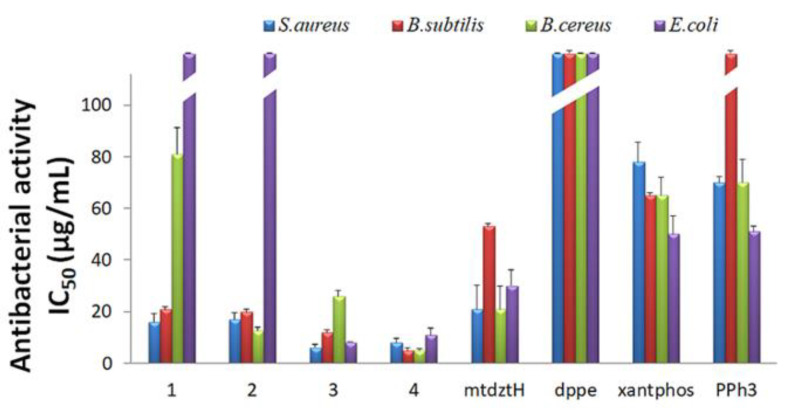
In vitro antibacterial activity of compounds **1**–**4**, and their ligands mtdztH, dppe, xantphos, and PPh_3_ in free form, expressed as half-minimum inhibitory concentration (IC_50_) values (in μg/mL) provided by a nonlinear curve fit-growth/sigmoidal-dose response on the experimental optical density data. Values are expressed as mean ± standard deviation (SD) of three replicate measurements (with the exception of values higher than 100 μg/mL).

Given the vast variety of Gram-(+) and Gram-(–) bacteria being responsible for severe infectious diseases nowadays, while showing resistance towards a broad spectrum of antibiotics or multi-drugs, there is an urgent need for the development of new and efficient antibacterial agents. In response to this imminent threat, Ag(I)-based compounds appear to be promising alternatives to common antibiotics, due to the ability of Ag(I) ions to interact with bacteria via various mechanisms and induce the latter’s growth inhibition without developing further resistance. In this study, we investigated the in vitro antibacterial activity of compounds **1**–**4** towards the Gram-(+) *S. aureus*, *B. subtilis, B. cereus*, and the Gram-(‒) *E. coli* bacterial strains. Their in vitro efficacy, as well as the efficacy of their respective ligands in free form, against the four bacterial strains was evaluated by the determination of their half-maximal inhibitory concentrations (IC_50_) and minimum inhibitory concentrations (MIC), and the results are presented in Table S4 and Figure 3.

As shown in Figure 3, generally, compounds **1**–**4** exhibited moderate-to-high activity against the studied bacteria, in contrast to their corresponding ligands in free form which displayed significantly lower antibacterial efficacy. Moreover, all compounds displayed higher antibacterial activity against the Gram-(+) compared to the Gram-(–) bacterial strains. Specifically, **3** and **4** were the most effective ones in terms of the growth inhibition of both Gram-(+) and Gram-(–) bacterial strains, showing relatively low IC_50_ and MIC values. In particular, **3** exhibited the lowest IC_50_ values of 8.1 μg/mL (9.5 ± 0.2 μΜ) and 6.1 μg/mL (7.1 ± 1.0 μΜ) against *E. coli* and *S. aureus*, whereas *B. subtilis* and *B. cereus* appeared to be more sensitive upon treatment with **4** with IC_50_ values of 4.9 μg/mL (4.5 ± 0.8 μΜ) and 5 μg/mL (4.6 ± 0.7 μΜ), respectively. Noteworthily, **1** and **2** showed negligible activity against the Gram-(–) *E. coli* bacteria, although they effectively inhibited the Gram-(+) bacterial growth with IC_50_ values ranging between 16 and 81 μg/mL. 

An explanation of the obtained experimental results can be provided on the basis of the individual structural characteristics of the four Ag(I) compounds and the different membrane architectures of the two types of bacteria. Generally, the majority of the 1,3,4-thiadiazole derivatives reported so far in the literature have been found to be able to efficiently penetrate the simply structured cell wall of the Gram-(+) bacteria, consisting of a single peptidoglycan layer, which is readily permeable to a range of different compounds, even highly hydrophobic ones [27]. On the contrary, the presence of the additional outer membrane of the Gram-(–) bacteria, consisting of lipopolysaccharides and porins, prevents the internalization of large hydrophobic compounds, while it selectively permits the internalization of cationic or small hydrophilic compounds. Based on the above, the high efficacy of [AgCl(mtdztH)(xantphos)] (**3**) against the Gram-(–) *E. coli* bacterial strain may be suggested to arise due to Cl^–^ dissociation (a process that might be facilitated in an aqueous biological environment) and the subsequent formation of a cationic Ag(I) complex that can interact effectively with the *E. coli* outer membrane, and ultimately leading to its accumulation in the interior of the bacterium. Similarly, the cationic oligomeric and/or monomeric species suggested to be formed by the dissociation of the polymeric compound **4** in solution can be effectively internalized into the studied Gram-(–) bacteria. Except from their total charge, another property that could be considered responsible for the observed differences in the antibacterial efficacy of the studied compounds, which is related to their ligands, is their lipophilicity. Generally, the presence of highly lipophilic ligands in a complex has a detrimental effect on its hydrophilicity/hydrophobicity balance that is necessary for effective antibacterial activity. Consequently, the lower antibacterial efficacy of **1** and **2** might be ascribed to the higher number of hydrophobic phenyl ring moieties of their PPh_3_ ligands compared to the lower number of phenyl substituents of the diphosphine ligands in **3** and **4**.

Comparison of the in vitro antibacterial efficacy of compounds **1**–**4** with a group of similar heteroleptic Ag(I) complexes bearing an analogous five-membered heterocyclic ring thioamide with an NH_2_-group substituent, i.e., 5-amino-1,3,4-thiadiazole-2-thiol (atdztH), that were recently reported, reveals the crucial role of the substituent groups of the thioamide heterocyclic ring in complexes’ antibacterial activity [20,21]. Narrowing our discussion to neutral Ag(I) complexes, the formerly presented [AgCl(atdztH)(xantphos)] was found to exhibit higher MIC and IC_50_ values than its isostructural [AgCl(mtdztH)(xantphos)] (**3**) against the same Gram-(+) bacterial strains, although slightly lower values against the same Gram-(–) bacteria. Similarly, the antibacterial efficacy of [Ag(atdtzt)(PPh_3_)_3_] was found to be slightly lower than that of the herein presented isostructural [Ag(mtdzt)(PPh_3_)_3_] (**2**). These findings suggest that replacement of the NH_2_ group of the 1,3,4-thiadiazole ring of the thioamide with a CH_3_ group is beneficial for high antibacterial activity for the particular group of Ag(I) complexes. Possibly, such a replacement results in a decrease in hydrophilicity and dipole moment of the complexes which, ultimately, facilitates their internalization into the bacterial membrane. Although general conclusions cannot be drawn (owing to the relatively small number of studied Ag(I) complexes), such a substituent-group replacement might be part of a general strategy for the enhancement of the antibacterial efficacy of Ag(I) complexes towards the development of novel, efficient and broad-spectrum antibacterial agents [28].

### 2.4. In Vitro Anti-Cancer Activity

Ag(I) complexes have recently emerged as promising anti-cancer agents. In our laboratory, we have investigated the anti-cancer activity of a number of Ag(I) complexes bearing a thioamide (or thioamidate) and phosphine ligands against different cancer cell lines and found to display significant bioactivity, with some of them also exhibiting high selectivity towards healthy cells [21,29]. Herein, we present the results of the in vitro evaluation of Ag(I) compounds **1**–**4** against the human cancer cell lines SKOV-3 (ovarian), Hup-T3 (pancreatic adenocarcinoma), DMS114 (lung), and PC3 (prostate), as well as the human normal cell line MRC5 (lung).

**Figure 4:**
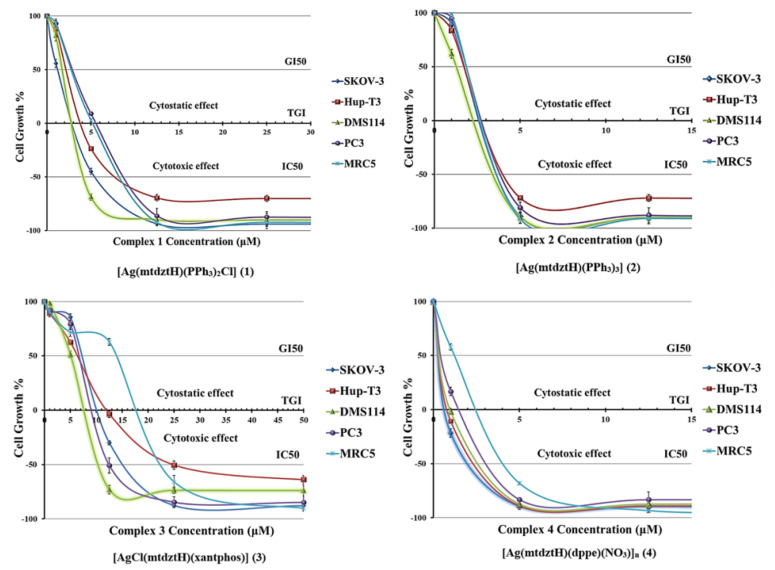
In vitro growth inhibition/cytostatic (GI_50_ and TGI, in μM) and cytocidal/cytotoxic (IC_50_, in μM) effects induced by compounds **1**–**4** against SKOV-3, Hup-T3, DMS114, PC3 human cancer, and MRC5 human normal cell lines (each point represents mean ± SEM of five measurements. Where error bar is not visible, its size is smaller than that of the corresponding symbol).

The growth inhibition/cytostatic and cytocidal/cytotoxic effects induced by **1**–**4** against cancer and normal cell lines are presented in Table S5 and reveal that all administered compounds caused the inhibition of cell proliferation in a dose-dependent manner. The dose–effect curves for all treated cell lines are illustrated in Figure 4, demonstrating the different chemosensitivity of the cell lines to the studied compounds. The order of potency of the compounds revealed to be the following: **4** > **2** > **1** > **3**, for all cell lines tested (except for MRC5 cells for which the order of **2** > **4** > **1** > **3** was documented), while the order of cell line chemosensitivity for all compounds was found to be: DMS114 > SKOV-3 > Hup-T3 = MRC5 ≥ PC3 (for **1**), DMS114 > PC3 > SKOV-3 > Hup-T3 = MRC5 (for **2**), DMS114 ≈ SKOV-3 = MRC5 ≥ PC3 ≈ Hup-T3 (for **3**), SKOV-3 > Hup-T3 ≈ DMS114 > PC3 > MRC5 (for **4**). Regarding the resulting chemosensitivity order, DMS114 cells were found to be apparently sensitive to the administration of **1**–**3**. However, the compound **4** exhibited higher efficacy against SKOV-3 cells in contrast to **1**–**3**.

All compounds displayed a very potent cytostatic and cytotoxic effect against the tested cell lines, displaying low IC_50_ values. However, **2** and **4** showed similar potency that was found to be three-to-six-fold higher than the corresponding pair of **1** and **3**. It is interesting to notice that the compound **4** exhibited the best anti-cancer activity among the tested compounds, whereas **1** and **3** displayed the least. In particular, **1** seemed to possess higher anti-cancer activity (a lower IC_50_ value) than its homologous complex **3**, indicating that the presence of the lipophilic PPh_3_ moieties plays an important role in the complex’s cellular uptake. Comparing **1** and **2** which are similar in structure (in **2,** a Cl atom is substituted by a Ph_3_P ligand), it was deduced that **2** exhibited higher activity, revealing the role of PPh_3_ ligand in affecting cell death. Meanwhile, the strong lipophilic character of these three PPh_3_ moieties had a pivotal role in the biological properties of **2**, enhancing its cytotoxic effect and causing both normal and cancer cell lethality at the same or lower concentrations. On the other hand, **3** and **4** demonstrated lower IC_50_ values against cancer cell lines compared to those found for the normal MRC5 cells, suggesting further investigation of their potential interaction with bio-macromolecule targets or carriers as promising anti-cancer agents.

Recently, we reported a group of similar Ag(I) complexes bearing phosphines and the (analogous to mtdztH) five-membered heterocyclic ring thioamide atdztH which also demonstrated significant in vitro anti-cancer activity but better selectivity against healthy cells than the herein presented compounds **1**–**4** [21]. Considering the similarity of the two groups of Ag(I) complexes with respect to their structures and ligands, it appears that the substituent group of the heterocyclic ring of their thioamide has a great impact on the observed anti-cancer activity. Possibly, the nature of the substituent group affects the hydrophilic or lipophilic character of the compounds altering, ultimately, their acceptability and cellular uptake. In this particular case, the less lipophilic Ag(I) complexes bearing the NH_2_-substituted thioamide exhibited better anti-cancer activity in a more selective manner than those containing the CH_3_-substituted thioamide.

### 2.5. In Vitro Antioxidant Activity

**Figure 5:**
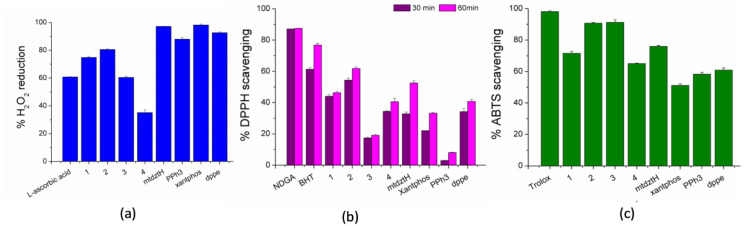
In vitro antioxidant ability of compounds **1**–**4**, as well as their ligands mtdztH, dppe, xantphos, and PPh_3_ in free form: (**a**) H_2_O_2_ reducing ability (% H_2_O_2_), (**b**) DPPH scavenging activity (% DPPH) and (**c**) ABTS scavenging activity (% ABTS).

There is a growing interest in developing novel synthetic chemicals with antioxidant properties to combat the free radicals’ pathological effects on eukaryotic cells. In particular, free radicals are known to disturb the normal cellular functioning leading to degenerative diseases such as Alzheimer’s disease and cancer, and, therefore, antioxidants attract much attention for the prevention or treatment of such disorders. Although a great number of metal-based compounds exhibiting significant antioxidant activity have been reported in the literature, hardly any Ag(I) compounds have been investigated. Having already established the high antibacterial and anti-cancer potential of compounds **1**–**4**, we proceeded to the investigation of their in vitro antioxidant activity. It should be mentioned that 1,3,4-thiodiazole-derivative thioamides in their free form have shown in vitro potential antioxidant activity, while scavenging free radicals at low concentrations [30]. Studies included the evaluation of the ability of **1**–**4** to scavenge DPPH and ABTS free radicals and cause the reduction of H_2_O_2_ (Table S6, Figure 5). For comparison purposes, antioxidant studies of mtdztH and phosphine ligands in their free form, as well as four selected reference compounds (NDGA, BHT, Trolox and L-ascorbic acid), are also included.

Hydroxyl radicals •OH and superoxide anion O_2_^•–^ represent the most important oxidants produced by H_2_O_2_, which are responsible for increasing DNA damage leading to cancer development and metastasis [31]. Therefore, compounds exhibiting strong ability of oxygen-based radical scavenging can be considered as promising anti-cancer agents. In Figure 5a, the high ability of **1**–**4** and their respective ligands to cause H_2_O_2_ reduction is depicted. As observed, **2** was found to be the most effective of all the compounds, inducing 80% H_2_O_2_ reduction which was higher than the reference L-ascorbic acid.

A DPPH assay is frequently used for the evaluation of a compound’s antioxidant capacity which is attributed to its ability to act as H^+^ or electron acceptor. The scavenging ability of the studied compounds is related to their potential anti-cancer and anti-inflammatory activity. Generally, **1**–**4** and their corresponding ligands displayed a moderate scavenging effect on DPPH radicals that was in most cases time-dependent, as shown in Figure 5b. Among the four Ag(I) compounds, **2** displayed the highest DPPH-scavenging ability which was, however, lower than that of the reference compounds BHT and NDGA.

Finally, the results of the ABTS assay revealed the total antioxidant activity of the compounds presented herein. Compounds **2** and **3** exhibited the strongest efficacy, although their ABTS-scavenging percentages appeared to be lower than that of the reference compound Trolox (Figure 5c).

### 2.6. CT DNA Interaction

DNA is recognized as one of the most common bio-macromolecular targets and several metal-based complexes, such as cisplatin, exert their anti-cancer effect by binding strongly to DNA [32]. However, many anti-cancer drugs interact with DNA in a non-covalent mode, which renders them less cytotoxic (at least through the particular mechanism) as their interaction occurs in a reversible manner [33]. Herein, we examine the binding mode and the strength of the binding of compounds **3** and **4** (which were found to be less toxic against normal cells, with respect to the studied cancer cells, and, therefore, more interesting as promising anti-cancer agents) with CT DNA.

An initial evaluation of the potential of the two compounds to intercalate in CT DNA was carried out by monitoring the changes in particular absorption bands in the UV–visible absorption spectra of **3** and **4** in their DMSO solutions, upon the addition of increasing amounts of CT DNA. In the case of **3**, an intense absorption band at 275 nm (due to ILCT transition) undergoes moderate hyperchromism up to 15% followed by a slight red-shift (bathochromism), upon addition of CT DNA (Figure S5b). On the contrary, the intra-ligand absorption band located at 272 nm in the spectrum of **4** showed an intensity decrease up to 13% (hypochromism) (Figure S5c). These data suggest that **3** might cause damage of the hydrogen bonds between DNA base-pairs whereas **4** would be able to adopt an intercalating binding mode. It should be pointed out that the observed spectral changes indicate the formation of complex–DNA conjugates, although they do not provide adequate information about the exact binding mode of the compounds on the DNA. Nevertheless, they allow the calculation of the binding constants K_b_, using the Wolfe–Shimer equation (Equation (1)) and the respective plots (Figure S6), which give an estimate of the strength of the complex–DNA interaction (Table 3). In general, K_b_ values for **3** and **4** fell in the 10^4^ − 10^5^ M^−1^ range, suggesting a relatively strong interaction with CT DNA. Moreover, the magnitude of the K_b_ values was of similar order of the corresponding binding constants found for recently reported complexes, i.e., [Ag(phimtH)_2_(dppe)]_n_(BF_4_)_n_ (phimtH = phenyl-imidazole-2-thione) [18,34].

**Figure 6:**
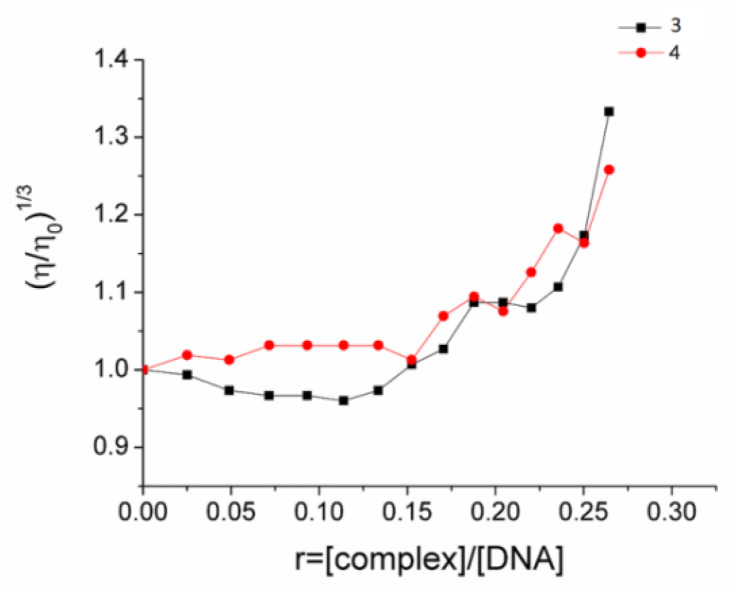
Relative viscosity (η/η_o_)^1/3^ changes in a CT DNA (0.1 mM)-buffered solution (150 mM NaCl and 15 mM trisodium citrate at pH 7.0) upon addition of increasing amounts of compounds **3** and **4** versus *r =* [compound]/[DNA].

Viscosity measurements provided a useful supplement to the UV–visible spectroscopy method described above to gain an insight into the interaction of the Ag(I) complexes with DNA. Given the fact that the relative viscosity of DNA is sensitive to changes in its length, the addition of a potential DNA binder to a solution of DNA is anticipated to affect its viscosity and, hence, may reveal details of the mode of interaction. In general, a significant increase in DNA viscosity may occur upon the intercalation of a compound between its base pairs, whereas a decrease or retention of the initial viscosity values could be assigned to groove binding or electrostatic interaction of the compound and the double helix. Herein, the relative DNA viscosity remained almost unchanged in the presence of low concentrations of **3** and **4** (up to *r* = 0.15), whereas a moderate increase in the viscosity was observed upon the addition of higher concentrations (*r* > 0.15) (Figure 6). These results, in combination with the nonplanar geometry of **3** and **4**, indicate that the complexes interact with DNA in a non-intercalating binding mode at low concentrations and probably intercalate in the double helix when *r* > 0.15.

**Figure 7:**
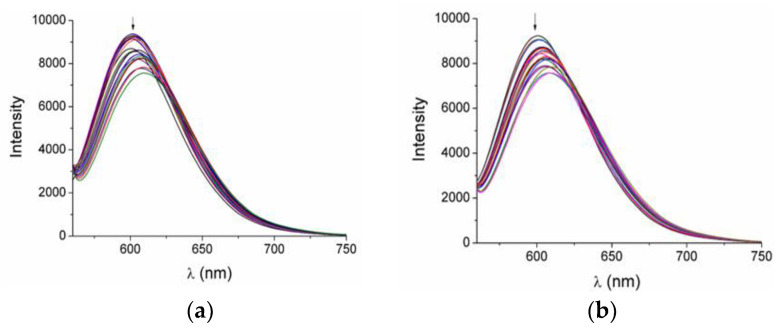
Fluorescence emission spectra (*λ*_ex_ = 540 nm) for EB–DNA ([EB] = 20 μM, [DNA] = 25 μM) in buffer solution upon addition of increasing amounts of compounds **3** (**a**) and **4** (**b**) The arrows show the changes in the emission intensity upon increasing amounts of the two compounds.

Ethidium bromide (EB) is a common DNA intercalator used in competitive studies for investigation of the potential ability of a compound to intercalate between DNA base-pairs. Within the context of the present work, increasing amounts of **3** and **4** were added to a buffered solution of EB–DNA conjugate and the changes in their emission spectra were recorded. The EB–DNA solution exhibited an intense emission band at *λ*_max_(em) = 592 nm, upon excitation at 540 nm. The addition of **3** and **4** to the EB–DNA solution led to the quenching of this EB–DNA emission band by ~26% (at low concentrations, *r* = 0.10-0.25) (Figure 7). Considering that **3** and **4** did not show emission maxima in this particular wavelength region, the observed fluorescence decrease can be attributed to the competitive behavior of compounds **3** and **4** with EB, while the low quenching percentage value gives an indication of the low capability of the compounds to replace EB in its binding sites on DNA. Fitting of these data on Stern–Volmer equation (Equation (2)) and the corresponding plots (Figure S7) allowed the estimation of the K_SV_ constants for qualification of the intercalating ability of the complexes. These were found to be of the order of 10^4^ M^−1^, which reveals relatively low binding strengths for both complexes. EB–DNA quenching constants k_q_ were also calculated (Equation (3)) and found to be >10^10^ M^−1^s^−1^ (Table 4), suggesting that the decrease in EB–DNA fluorescence induced by these two compounds occurs via a static mechanism due to the formation of a new compound–DNA conjugate [35].

In conclusion, compounds **3** and **4** have the ability to bind to CT DNA but with different strength and binding modes than the previously reported **1** and **2** (which demonstrate high affinity to CT DNA adopting an intercalation-binding mode). In particular, their relatively low binding constants as well as their low ability to change DNA viscosity and effectively displace EB are in agreement with their low efficacy to intercalate between base pairs of the double-stranded DNA. Considering the structural features of **3** and **4**, and in particular their ability to form cationic entities in aqueous media and the low number of phenyl group substituents on their ligands compared to **1** and **2**, a nonclassical intercalation mode (e.g., electrostatic interactions) is more probable to occur at low concentrations, with intercalation becoming also probable at higher concentrations.

### 2.7. Albumin-Binding Studies

Serum albumins (SA) are important drug carriers in the circulatory system and they are responsible for drug transportation and distribution [36,37]. To gain an idea about the pharmacokinetic properties of compounds **3** and **4**, the more interesting compounds as promising anti-cancer agents, related to their absorption and distribution, we investigated their ability to interact with BSA and HSA [38].

**Figure 8:**
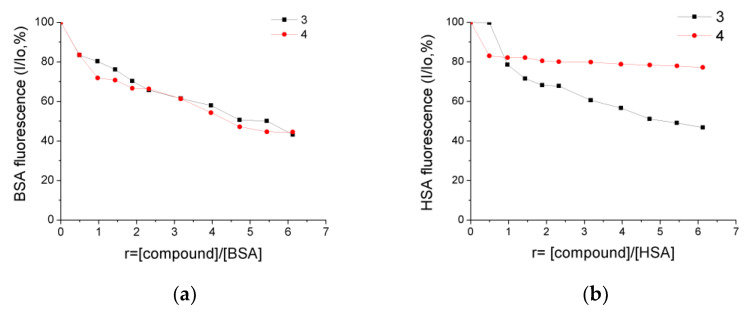
Plots of percentage of fluorescence intensity changes (I/I_o_, %) at (**a**) λ_max_(em) = 350 nm for BSA and (**b**) λ_max_(em) = 338 nm for HSA, in buffer solutions (150 mM NaCl and 15 mM trisodium citrate at pH = 7.0) upon addition of increasing amounts of compounds **3** and **4** versus r = [compound]/[BSA] and r = [compound]/[HSA], respectively.

The BSA and HSA buffer solutions displayed high-intensity emission bands with λ_max_(em) at 350 nm and 338 nm, respectively, upon excitation at 295 nm. The addition of increasing concentrations of **3** and **4** to the SA solutions led to quenching of the corresponding emission bands, indicating their binding to SA is followed by conformational changes in the proteins’ structure. In detail, a moderate decrease in BSA fluorescence of 56.8% and 44.5% was observed upon the addition of **3** and **4**, respectively (Figure 8a). Under similar experimental conditions, the corresponding HSA emission quenching reached the values of 53.3% and 23% for **3** and **4**, respectively (Figure 8b). 

The fluorescence-quenching constants k_q_ for SA, obtained using the Stern–Volmer equation (Equation (4)) and the corresponding plots (Figures S8 and S9), were found to fall in the range of 10^11^-10^12^ M^−1^s^−1^ (Table 5), suggesting a static emission-quenching mechanism due to the potential formation of new conjugates between the complexes and the albumins. The SA-binding constants K of **3** and **4** were calculated using the Scatchard equation (Equation (5)) and the corresponding plots (Figures S10 and S11). Their values are of the order of 10^4^–10^5^ M^−1^ (Table 5) which indicate a rather tight but reversible non-covalent binding of the two compounds to BSA and HSA, suggesting their successful transport and deposition to targeted action sites (compared to the K values of 10^15^ M^−1^ calculated for the strongest non-covalent interaction of various ligands to avidin).

Therefore, it can be concluded that compounds **3** and **4** can interact strongly but reversibly with BSA and HSA, as revealed by their quenching abilities and the respective binding constants. Interestingly, the emission-quenching data revealed a superior quenching ability of **3** compared to **4**, which might be related to their binding to different sites of BSA and HSA. Further studies are currently being conducted by our group aiming to elucidate the exact mechanism of interaction of the complexes with serum albumins.

### 2.8. Molecular Docking Calculations

With a view to elucidate the in vitro activity of the complexes, shedding light on their possible anti-tumor activity, a target therapeutic protein expressed in all four cell lines was chosen for in silico studies. A common protein involved in cancer growth overexpressed in the tested cancer cells is the fibroblast growth factor receptor (FGFR) preceding in the majority of intracellular signal transduction pathways. Fibroblast growth factors (FGFs) have been implicated in various aspects of cellular responses in both normal and malignant cells, including malignant transformation, tumor mitogenesis, angiogenesis, and chemoresistance. Specifically, FGFRs play essential roles in mediating cell proliferation, migration, and survival [39]. The FGFR family consists of four highly conserved transmembrane receptor tyrosine kinases (RTKs) (FGFR1–4) and their aberrant activation gives rise to the activation of many cancer-related pathways, such as MAPK and JAK/STAT [40], a fact that ultimately accelerates malignancy in cancer [41]. FGFR1, when bound to a proper FGF, elicits cellular responses by activating signaling pathways that include the: (a) Phospholipase C (PLCγ)/PI3K/AKT, (b) Ras subfamily/ERK, (c) Protein kinase C, (d) IP3-induced raising of cytosolic Ca^2+^, and e) Ca^2+^/calmodulin-activated elements and pathways [42]. 

DMS114 has mainly FGFR1 with a small amount of FGFR3 expression [43]. It has been shown that BGJ398 compound inhibits FGFR signaling with the levels of total FGFR1/FGFR3 and FGFR substrate 2 (FRS2) being significantly decreased in a dose-dependent manner. Previous studies have shown that the FGFR inhibitor PD173074 potentiated the effects of cisplatin in small-cell lung cancer, thus showing by a different mechanism that the inhibition of FGFRs can augment the effects of cisplatin [44].

Similarly, SKOV3 cells were also found to express FGFR1 [45] along with FGFR2, 3, and 5. This study showed a reduction in the expression of FGFR2, FGFR3, FGFR4, FGF3 and FGF7 using shRNAi, significantly inhibiting SKOV3 ovarian cancer cell line proliferation by 40–80%. The inhibition of FGFR2 and/or FGF3 and FGF7 impacted significantly on SKOV3 proliferation and reduced significantly the IC_50_ of cisplatin in vitro. 

Furthermore, FGFRs have been found to represent an important therapeutic target in the fight against pancreatic ductal adenocarcinoma (PDAC). In pancreatic cancer, aberrations in the FGFR pathway, particularly FGFR1 overexpression, have been reported. The clinical significance of FGFR1 expression in pancreatic cancer has been recently investigated [46] showing that the FGFR1-based subclassification of pancreatic cancer may lead to new therapeutic approaches for the FGFR1-positive subtype. Recently, a study on the inhibition of FGF/FGFR signaling on this very aggressive form of cancer in vitro showed that knocking down FGFR1 and FGFR2 decreased their tumorigenesis abilities in vivo [47]. Deregulation of the FGF/FGFR axis is involved in oncogenesis, tumor progression and resistance to anticancer treatment across multiple types of tumors [48]. Over-expression of FGFs and their receptors is a feature of pancreatic cancer and correlates with poor prognosis [49] In a previous study, the inhibition of FGFR2 attenuated the proliferation and invasion of pancreatic cancer [50]. In this study, FGF1, FGF2, FGF5, and FGF7 were found to be overexpressed in PDAC. FGFRs may thus constitute novel therapeutic targets for PDAC. 

Additionally, the elucidation of the role of FGFR1 induction in acquired resistance to MET and VEGFR2 inhibition by cabozantinib in prostate cancer (PCa) was studied recently [51]. The researchers concluded that the FGFR1 overexpression mediated acquired resistance to MET/VEGFR2 inhibition, leveraging this understanding to improve therapy outcomes. They revealed that the molecular basis of resistance to MET inhibition in prostate cancer is FGFR1 activation through a YAP/TBX5-dependent mechanism. In other studies, it was found that PCa cells with high FGFR1 expression increased the bone metastatic progression of the PCa and that FGFR1 accelerates PCa metastatic dissemination [52] and FGFR blockade with dovitinib (TKI258) has clinical activity in a subset of men with castration-resistant PCa (CRPC) and bone metastases [53]. These findings suggest that targeting FGFRs has therapeutic activity in advanced PCa and provide direction for the development of therapies with FGFR inhibitors.

**Figure 9:**
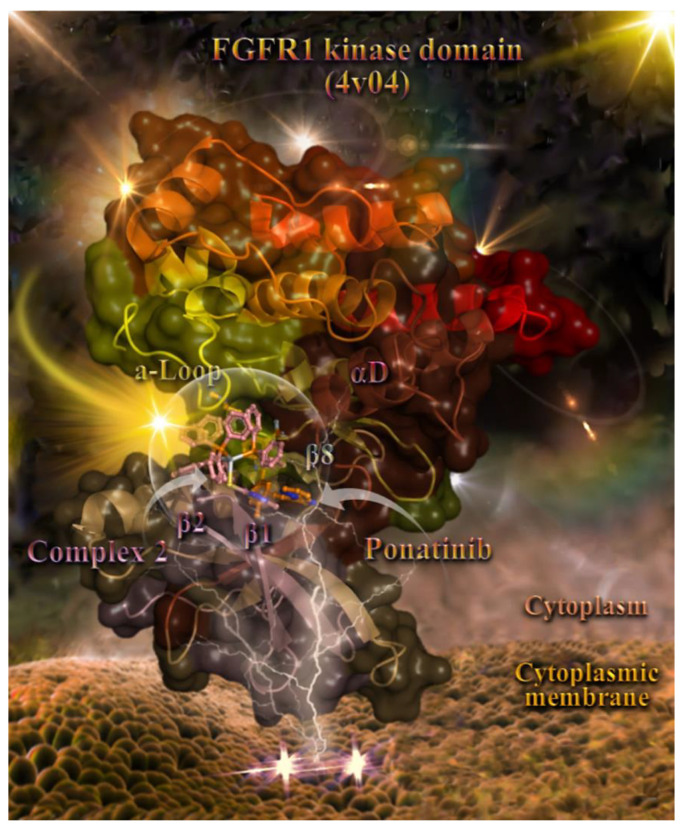
Docking pose orientation of compound **2** in the crystal structure of the catalytic domain of fibroblast growth factor receptor 1 (FGFR1) kinase in a complex with its inhibitor drug ponatinib (PDB accession number: 4V04). FGFR1 is localized in the cytoplasmic membrane towards the intracellular cytoplasm (transmembrane and extracellular domains are not shown). The target protein is illustrated as a cartoon and the docked **2** as well as the superimposed co-crystallized inhibitor ponatinib are rendered in ball-and-stick mode and colored according to atom type in salmon and orange, respectively. FGFR1 protein’s segments: activation loop (a-Loop); aD helix; and β1, β2, and β8 antiparallel beta sheets forming the binding pocket of **2** are depicted in the structure colored according to cartoon (split pea green, chocolate, salmon, salmon, and split pea green, respectively). A-loop is depicted in its active (open) conformation. Complex **2** is found adjacent to ponatinib at the ATP-binding cleft of the protein. Heteroatom color-code: O: red, N: blue, S: yellow, P: orange, and Ag: gray. Hydrogen atoms are omitted from all molecules for clarity. The final structure was ray-traced and illustrated with the aid of PyMol Molecular Graphics Systems.

In order to elucidate the anti-cancer activity of **1**–**4** on different human cancer cells, we adopted molecular docking studies on FGFR1 target protein. The best anti-cancer activity induced by **1**–**4** against cancer and normal cell lines, revealed to be that of **2** and **4** exhibiting the highest growth inhibition and cytostatic and cytocidal/cytotoxic effects (Table S5). It is of interest to attempt a possible correlation of this activity with the inhibition potency of the compounds on FGFR1. However, due to difficulties in docking calculations of polymeric compounds such as **4**, we restricted our in silico studies only to **2**. The binding of **2** in the catalytic site of the FGFR1 kinase domain is depicted in Figure 9. The Glide Standard Precision (SP) binding energy ΔG_bind_ of **2** docked on FGFR1 (PDB accession number: 4V04) is −38.27 Kcal/mol. The computational process revealed that **2** was bound in the active catalytic site of FGFR1 kinase domain, at the same place occupied by the FGFR1 inhibitor drug ponatinib, and especially at the region of the ATP-binding pocket of the enzyme. Ponatinib occupied the cleft between the N- and C-terminal lobes where ATP would otherwise bind [54].

Complex **2** seems to be anchored in the binding pocket of the enzyme formed by the FGFR1 protein’s segments: the activation loop (a-Loop); aD helix; and β1, β2, and β8 antiparallel beta sheets. Ponatinib (flanked by β2, β3, and β5 sheets and also positioned between the loop formed by the β4 sheet and αC helix and also the hinge region connecting the β5 sheet and αD helix) and **2** are flanked by an a-Loop and β1 and β2 beta sheets, in the upper and lower part of the binding cavity, respectively, and also by the hinge region connecting the β5 sheet and αD helix (in the middle position of the binding cleft). 

**Figure 10:**
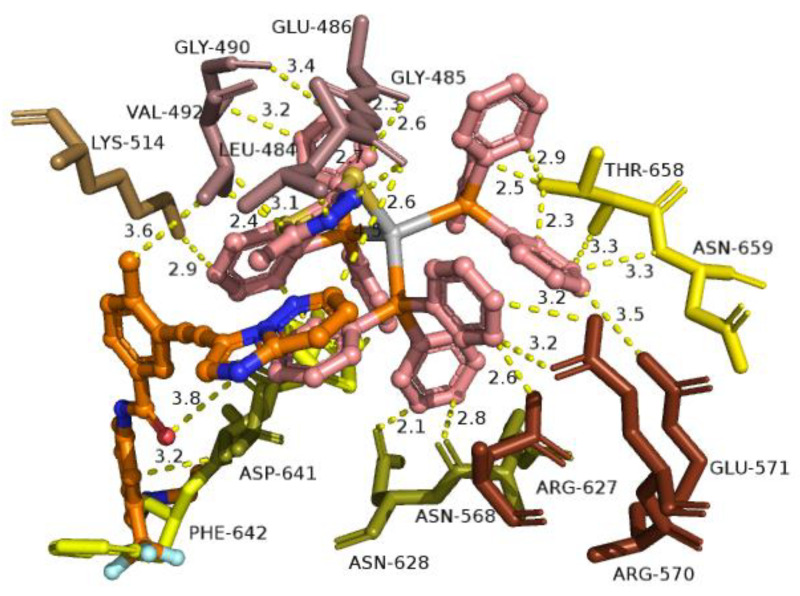
A close-up view of the binding site mapping architecture of the best binding pose of complex **2** in the crystal structure of FGFR1 target protein. Complex **2** is illustrated to be anchored at the ATP-binding cleft of the protein at the same place as the co-crystallized inhibitor ponatinib. Docked complex **2** as well as the superimposed ponatinib are rendered in ball-and-stick mode and colored according to atom type in salmon and orange C atoms, respectively. Selected contacting residues of the binding pocket are colored according to the FGFR1 protein’s segments shown in Figure 9. Binding contacts are shown as yellow dotted lines. Heteroatom color-code: O: red, N: blue, S: yellow, P: orange, and Ag: gray. Hydrogen atoms are omitted from all molecules for clarity. The final structure was ray-traced and illustrated with the aid of PyMol Molecular Graphics Systems.

A close-up view of the binding site mapping architecture of the best binding pose of complex **2** in the crystal structure of FGFR1 target protein depicting the binding interactions with the residues of the ATP-binding cleft of the protein is illustrated in Figure 10.

The ligand-binding contacts of **2** in the active site of the kinase domain include Asn N568 (π-polar, 2.6 Å), Arg R570 (π-cation, 3.5 Å), and Glu E571 (π-anion, 3.2 Å) of the αD helix; Gly G490 (π-polar, 3.2 Å) at the starting point of the β2 sheet; Leu L484 (π-polar, 2.9-3.9 Å; hydrophobic, 3.4 Å; π-alkyl, 2.7 Å) and Gly G485 (hydrogen bond, 2.5 Å; π-alkyl, 3.6 Å; polar, 3.4 Å) of the terminal region of the β1 sheet; Glu E486 (π-anion, 2.6 Å; π-alkyl, 2.0-2.3 Å; π-polar, 2.8 Å and 3.4 Å) of the loop following the β1 sheet; Phe F642 (π-π T-shaped, 2.9 Å; π-π displaced type, 2.6 Å; π-polar, 2.1 Å; π-alkyl, 2.4 Å), Thr T658 (π-polar, 2.5 Å; π-alkyl, 2.5–3.0 Å), Asp D641 (π-anion, 2.6 Å), and Asn N659 (π-polar, 3.3 Å; π-alkyl, 3.7 Å) of the activation loop (a-Loop); Arg R627 (π-polar, 2.3 Å; π-alkyl, 2.7 Å and 3.3 Å) of the loop connecting the β8 sheet with the αE helix; Lys K514 (π-cation, 2.9 Å) and Val V492 (π-alkyl, 2.4–3.0 Å) of the β2 sheet; and Asn N628 (π-polar, 2.1 Å). Among the binding contacts of **2**, three residues seem to be in common with those of the binding of ponatinib, namely, Phe (F642), Leu (L484), Asp (D641), and Val (V492). The phenyl ring of one of the PPh_3_ ligands of **2**, being in contact to the F642 residue, appears also to be in close proximity to the imidazopyridazine ring of ponatinib which also makes contact with F642. Notably, the F642 residue belongs to the activation loop (a-Loop) of the protein. The other two residues, L484 and V492, are part of beta strands β1 and β2, respectively. One of the PPh_3_ ligands of **2** extends away from the hinge region making limited interactions with the enzyme, involving four binding contacts with threonine T658 and asparagine N659 of the a-Loop, glutamate E486 of the loop following the β1 sheet, and arginine R570 of the αD helix. The critical binding contact of ponatinib, F642, seems to be also one of the most important contacts of **2**, stabilizing its molecule in the active site by π-π displaced (offset) and also π-π T-shape with two phenyl rings of a PPh_3_ ligand. One of the phenyl rings is found in close proximity to the imidazopyridazine ring of ponatinib that mediates its binding to F642 via also a π-π displaced-type interaction, sharing common binding contacts with it. The rest of the binding contacts of ponatinib (A512, E531, M534, M535, I538, I544, I545, V559, Y563, A564, G567, C619, I620, H621, R622, L630, D641, and L644) do not seem to be involved in direct interactions with **2**, thus indicating a more stable binding of ponatinib. Further stabilization of **2** in the catalytic active site of the kinase is achieved by the interaction of one of its PPh_3_ ligands with the proximal end of the αD helix via residues N568 and E571. Interestingly, the in silico study results demonstrating the ability of **2** to bind to the bio-macromolecule FGFR1 were found to be in accordance with in vitro studies, contributing to the interpretation of the biologic activity of **2**. 

## 3. Conclusions

In this work, we report a series of Ag(I) compounds bearing the CH_3_-substituted thiadiazole-based thioamide 5-methyl-1,3,4-thiadiazole-2-thiol (mtdztH) and phosphines, i.e., [AgCl(mtdztH)(PPh_3_)_2_] (**1**), [Ag(mtdzt)(PPh_3_)_3_] (**2**), [AgCl(mtdztH)(xantphos)] (**3**), and [Ag(mtdztH)(dppe)(NO_3_)]_n_ (**4**), and the assessment of their in vitro antibacterial and anti-cancer efficiency. Compounds **3** and **4** bearing diphosphine co-ligands were found to exhibit stronger antibacterial activity than the PPh_3_-containing compounds, as well as broad-spectrum antibacterial activity characteristics, against both Gram-(+) and Gram-(–) bacteria, with IC_50_ values as low as 4.6 μΜ. Effective in vitro cytotoxic activity was observed for all compounds against human ovarian, pancreatic, lung, and prostate cancer cell lines, with **3** and **4** maintaining the normal fibroblast cells’ viability at relatively higher levels. Compounds **1**–**4** displayed a moderate ability to scavenge free radicals, as well as high affinity to interact with CT DNA through nonclassical intercalation modes, and tight but reversible binding on serum albumins, suggesting the likely implication of these properties in their bioactivity mechanisms. Finally, molecular docking calculations shed light on the in vitro anti-cancer activity of the compounds, suggesting a possible role on the FGFR1 target protein, affecting cancer cells’ functionalities.

In summary, considering the herein-presented bioactivity results in combination with those obtained for our recently reported Ag(I) complexes bearing small-size heterocyclic ring thioamide ligands with different ring substituents, i.e., CH_3_- vs. NH_2_ and CF_3_ groups, one can recognize the great importance of the thioamide substituent group on the bioactivity of the respective complexes. Pertaining to their antibacterial activity, in particular, the replacement of the NH_2_ substituent group of the thioamide ligand by a CH_3_ group appears to induce a higher efficacy to the respective Ag(I) complexes, possibly by facilitating their internalization into the bacterial cells. These results can be utilized as part of a general strategy for the enhancement of the antibacterial efficacy of these Ag(I) complexes towards the development of novel, efficient, and broad-spectrum antibacterial agents.

## 4. Materials and Methods

### 4.1. General Procedures and Chemicals

All manipulations were carried out under atmospheric conditions, unless otherwise mentioned. Solvents were purified according to established methods and allowed to stand over molecular sieves for 24 h. Silver nitrate (AgNO_3_), silver chloride (AgCl), silver tetrafluoroborate (AgBF_4_), 5-dimethyl-1,3,4-thiadiazole-2-thione (mtdztH), thriphenylphosphine (PPh_3_), 4,5-bis(diphenylphosphano)-9,9-dimethylxanthene (xantphos), bis[(2-diphenylphosphino)phenyl] ether (DPEphos) were purchased from commercial sources and used as received.

For the antioxidant activity studies, H_2_O_2_, 2,2-diphenyl-1-picryl-hydrazyl-hydrate (DPPH), ABTS, butylated hydroxytoluene (BHT), 6-hydroxy-2,5,7,8-tetramethylchromane-2-carboxylic acid (trolox), nordihydroguaiaretic acid (NDGA) were obtained from commercial sources and used without any further purification. 

The in vitro biomolecule interaction studies were assessed using calf-thymus (CT) DNA, BSA (bovine serum albumin), HSA (human serum albumin), EB (ethidium bromide), NaCl, trisodium citrate and solvents which were of reagent grade and were used as purchased from commercial sources without any further purification.

For the cytotoxicity studies, four well-established human cancer cell lines (ovarian, lung, prostate, and pancreatic) and a human normal lung cell line were adopted to explore the anticancer activity of the studied compounds.

### 4.2. Syntheses

#### 4.2.1. [AgCl(mtdztH)(PPh_3_)_2_] (**1**)

AgCl (0.072 g, 0.5 mmol) was suspended in 25 mL of CH_3_CN and PPh_3_ (0.262 g, 1.0 mmol) was added in small portions. The resulting mixture was stirred at 50 °C for 24 h in dark and then an amount of 0.066 g (0.5 mmol) of mtdztH was added. The reaction mixture was further stirred at 60 °C for 2 h in dark and then it was allowed to cool at room temperature. After filtering the reaction mixture, the filtrate was set aside in dark to evaporate slowly at room temperature and, over a period of 24 h, crystals of **1** were grown which were collected. Yield: 0.152 g (38%). Anal. Calc. for [C_39_H_34_AgClN_2_P_2_S_2_]: C, 58.54; H, 4.28; N, 3.50. Found: C, 58.24; H, 4.52; N, 3.71%. FTIR (KBr, cm^−1^): 3044w, 2955w, 1552m, 1477vs, 1432vs, 1277vs, 1198 m, 1097 vs, 1049 vs, 1024 m, 998 m, 748 vs, 694 vs, 504 vs, 489 vs. ^1^H NMR (500 MHz, CDCl_3_): δ(ppm): 13.30 (s,1H, NH), 7.67-7.48 (m, 6H, o-H, PPh_3_), 7.44-7.40 (m, 6H, o-H, PPh_3_), 7.33-7.30 (m, 18H m-H, p-H, PPh_3_), 2.49 (s, 3H, -CH_3_). UV–Vis (CH_2_Cl_2_), λ/nm (log ε): 256 (4.42), 310 (3.51).

#### 4.2.2. [Ag(mtdzt)(PPh_3_)_3_] (**2**)

A portion of AgBF_4_ (0.097 g, 0.5 mmol) was dissolved in a 10-mL mixture of CH_3_CN:CH_3_OH (1:1 v/v) and then PPh_3_ (0.262 g, 1.0 mmol) was added in small portions. The resulting mixture was stirred at 50 °C for 20 min in dark and then a 20-mL methanolic solution of K^+^mtdzt^‒^ in the same solvent mixture, obtained from the deprotonation of the corresponding amount of mtdztH (0.066 g, 0.5 mmol) with 1.0 mL of 0.5 M solution of KOH in CH_3_OH, was added dropwise. After stirring at 65^o^C for 1 h, the resulting suspension was filtered in order to remove a small amount of an off-white solid. The filtrate was set aside in dark to evaporate slowly at room temperature and, after 5 days, crystals of **2** started growing, which were collected. Yield: 0.300 g (57%). Anal. Calc. for [C_58_H_52_AgN_2_OP_3_S_2_]: C, 65.85; H, 4.95; N, 2.65. Found: C, 66.01; H, 4.70; N, 2.83%. FTIR (KBr, cm^−1^): 3448br, 3052w, 1579m, 1481s, 1437vs, 1368m, 1307m, 1281w, 1179s, 1087s, 996s, 743vs, 698vs, 516vs, 500vs. ^1^H NMR (500 MHz, CDCl_3_): δ(ppm): 7.67-7.63 (3H, p-H), 7.57-7.47 (6H, p-H), 7.37-7.32 (18H, o-H), 7.30-7.28 (18H, m-H), 2.40 (s, 3H, -CH_3_). UV–Vis (CH_2_Cl_2_), λ/nm (log ε): 257 (4.56).

#### 4.2.3. [AgCl(mtdztH)(xantphos)] (**3**)

To a suspension of AgCl (0.043 g, 0.3 mmol) in 15 mL of dry CH_3_CN, xantphos (0.174 g, 0.3 mmol) was added in small portions, followed by stirring for 1h at 60℃ in dark. To the resulting mixture formed, a solution of mtdztH (0.040 g, 0.3 mmol) in 15 mL MeOH, was added dropwise. The reaction mixture was allowed to cool at room temperature and after filtration; the filtrate was set aside in dark to evaporate slowly. Over a period of 5 days, large colorless crystals of **3** were formed, which were collected. Yield 0.122 g (48%). Anal. Calcd. for [C_42_H_36_AgClN_2_OP_2_S_2_]: % C, 59.06; H, 4.25; N, 3.28. Found: % C, 59.42; H, 4.57; N, 3.57. FTIR (KBr, cm^−1^): 3445b, 2957m, 1472m, 1434m, 1403s, 1227s, 1054m, 875w, 748m, 695m, 507m. ^1^H NMR (500 MHz, CDCl_3_): δ(ppm): 13.30 (s,1H, NH), 7.54-7.52 (dd, 2H, xantphos), 7.38-7.34 (m, 8H, o-H, phenyl), 7.29-7.27 (m, 4H, p-H, phenyl), 7.22-7.19 (m, 8H, m-H, phenyl), 7.08-7.05 (t, 2H, xantphos), 2.47, (s, 3H,-CH_3_), 1.65(s, 6H, -CH_3_). UV-visible (CH_2_Cl_2_), λ/nm (log ε): 254 (4.21), 307 (4.01).

#### 4.2.4. [Ag(mtdztH)(dppe)_2_(NO_3_)]_n_ (**4**)

To AgNO_3_ (0.042 g, 0.25 mmol) suspended in 20 mL of CH_2_Cl_2_, dppe (0.100 g, 0.25 mmol) was added in small portions and the mixture was stirred for 30 min at room temperature in dark. Then, mtdztH (0.033 g, 0.25 mmol) was added and the reaction mixture stirred at room temperature for 1 h. After filtration, the filtrate was layered with n-hexane and after 5 days, large colorless crystals of **4** were grown, which were collected. Yield: 0.139 g (50%). Anal. Calc. for [C_29_H_28_AgN_3_O_3_P_2_S_2_]: % C, 49.72; H, 4.03; N, 6.00. Found: % C, 49.35; H, 3.74; N, 5.78. FTIR (KBr, cm^−1^): 3368 b, 3049 m, 1482 s, 1433 vs, 1339 s, 1273 w, 1191 w, 1179 w, 1097 s, 1018 m, 884 w, 740 s, 726 s, 693 vs, 510 s, 478 s, 451 w. ^1^H NMR (500 MHz, CDCl_3_) δ(ppm): 7.75–7.68 (m, o-H), 7.53–7.50 (m, m-H), 7.45–7.36 (m, p-H), 2.78 (s, -CH_3_), 2.52–2.51(dd, -CH_2_, dppe). UV-visible (CH_2_Cl_2_), λ/nm (log ε): 265 (4.49)

### 4.3. Instrumentation

Elemental analyses were obtained on a PerkinElmer 240B elemental microanalyzer. Infra-red spectra were recorded on a Nicolet FT-IR 6700 spectrophotometer as KBr discs in the region of 4000–400 cm^−1^. UV-visible electronic absorption spectra and emission/excitation fluorescence spectra were recorded on a Hitachi F-7000 fluorescence spectrometer both in the solid state and in dilute organic solvent solutions. ^1^H NMR spectra were recorded on an Agilent 500 spectrometer using deuterated solvent solutions, while chemical shifts were reported as δ values using the solvent as internal standard.

### 4.4. X-ray Crystal Structure Determinations

Single crystals of all compounds, suitable for X-ray diffraction analysis, were mounted on thin glass fibers with the aid of an epoxy resin. X-ray diffraction data were collected on a Bruker Apex II CCD area-detector diffractometer, equipped with a Mo Ka (λ = 0.71070 Å) sealed tube source, at 295 K, using the φ and ω scans technique. The program Apex2 (Bruker AXS, 2006) was used in data collection, cell refinement, and data reduction [55]. Structures were solved and refined with full-matrix least-squares using the program Crystals [56]. Anisotropic displacement parameters were applied to all non-hydrogen atoms, while hydrogen atoms were generated geometrically and refined using a riding model. Details of crystal data and structure refinement parameters are shown in Table 1. Plots of the molecular structures of all compounds were obtained by using the program Mercury [57].

### 4.5. In Vitro Antibacterial Activity

The in vitro antibacterial activity of **1**–**4** and their corresponding ligands towards *Escherichia coli* (*E. coli*) and *Bacillus subtilis* (*B. subtilis*), *Bacillus cereus* (*B. cereus*) and *Staphylococcus aureus* (*S. aureus*) bacterial strains was evaluated using a method of using progressive double dilutions in MMS contained the concentrations of 100, 50, 25, 12.5, and 6.25 μg/mL of the compounds in DMSO [58]. The growth of bacteria was estimated by measuring the turbidity of the culture, as previously reported [59]. Two cultivation media used for antibacterial activity tests were: (i) the Luria-Bertani broth [1% (w/v) tryptone, 0.5% (w/v) NaCl and 0.5% (w/v) yeast extract] and (ii) the minimal medium salts broth containing [1.5% (w/v) glucose, 0.5% (w/v) NH_4_Cl, 0.5% (w/v) K_2_HPO_4_, 0.1% (w/v) NaCl, 0.01% (w/v) MgSO_4_·7H_2_O]. The pH of the media was adjusted to 7.0. 

### 4.6. In Vitro Anticancer Activity

#### 4.6.1. Cell Culture

Compounds **1**–**4** were tested for their in vitro anticancer activity against four well-established human cancer cell lines (ovarian, lung, prostate, and pancreatic) and a human normal lung cell line. Ovarian cancer (SKOV-3) cells, small cell lung cancer (DMS114) cells, prostate adenocarcinoma (PC-3) cells, pancreatic adenocarcinoma (HuP-T3) cells, and normal lung (MRC5) cells, were tested for cytostatic (growth inhibition: IC_50_, TGI) and cytotoxic/cytocidal (IC_50_) activity against the tested compounds at concentrations of 0.5-100 µМ. The cell lines were obtained from the American Type of Culture collection (ATCC), except HuP-T3 which was kindly donated from University of Crete, School of Medicine, Greece, and were grown in different culture medium according to the instructions. Cultures were maintained at 37 °C in 5 % CO_2_ and 95 % air.

#### 4.6.2. In Vitro Cytotoxicity Assay

For the determination of the cell viability, it was employed the MTT assay. Cells were plated in 96-well plate at a density of 1 × 10^4^ cells/mL per well and maintained for 72 h at 37 °C in a 5% CO_2_ incubator and grown as monolayers. After 24 h, cells were treated with 0.5–150 μM of the tested compounds for 48 h. The viability of cultured cells was estimated by a (3-(4,5-imethylthiazol-2-yl)-2,5-diphenyltetrazolium bromide (MTT) metabolic assay as previously described [60,61,62].

In brief, MTT (Sigma, St Louis, Missouri, USA) was dissolved in PBS in a concentration of 5 mg/mL, filter sterilized, and stored at 4 °C. Then, 50 μL of stock solution was added to each culture and incubated for 3 h at 37 °C. Formazan crystals were solubilized by DMSO (100 μL). Absorbance of the converted dye was measured at a wavelength of 540 nm on ELISA reader (VersaMax Microplate Reader Orleans, USA). The mean concentrations of each drug that generated 50% or total (100%) growth inhibition (GI_50_ and TGI, respectively) as well as the drug concentrations that produced cytotoxicity against 50% of the cultured cells [half maximal inhibitory concentration (IC_50_)] were calculated using the linear regression method [61]. Using seven absorbance measurements [time 24 h (Ct24), control growth 72 h (Ct72), and test growth in the presence of drug at five concentration levels (Tt72x)], the percentage of growth was calculated at each level of the drug concentrations. The percentage growth inhibition was calculated according to National Cancer Institute (NCI) as: {[(Tt72x) − (Ct24)]/[(Ct72) − (Ct24)]} × 100 for concentrations for which Tt72x>Ct24 and {(Tt72x) − (Ct24)]/(Ct24)} × 100 for concentrations for which Tt72x<Ct24. The GI_50_ was calculated from {[(Tt72x) − (Ct24)]/[(Ct72) − (Ct24)]}×100 = 50 while the TGI from {[(Tt72x) − (Ct24)]/[(Ct72) − (Ct24)]} × 100 = 0, and IC_50_ from {[(Tt72x) − (Ct24)]/(Ct24)} × 100 = 50. All experiments were carried out in triplicate. A significant difference was presumed to exist when *p* ≤ 0.05 (two tailed paired *t* test).

### 4.7. In Vitro Antioxidant Activity

In vitro antioxidant activity studies for **1**–**4** and their free ligands were performed in order to determine their ability to scavenge DPPH, and ABTS free radicals and to reduce H_2_O_2_ and was compared with the antioxidant capacity of reference compounds (NDGA, BHT, trolox and ascorbic acid). All measurements were carried out in triplicate and the standard deviation of absorbance was <10% of the mean.

#### 4.7.1. Reduction of Hydrogen Peroxide 

The antioxidant activity of **1**–**4** and their corresponding ligands (atdztH, mtftH PPh_3_, xantphos, DPEphos) in free form against hydrogen peroxide was determined by monitoring the reduction of H_2_O_2_. The reaction mixture contained 20 μL of each of the tested compounds (0.1 mM) and 5 μL H_2_O_2_ solution (40 mM) in phosphate buffer (50 mM, pH 7.4). The absorbance was measured at 230 nm after 20 min. The antioxidant activity of the compounds was expressed as percent reduction of H_2_O_2_ (%H_2_O_2_) [63]. Ascorbic acid (or vitamin C) was used as an appropriate standard.

#### 4.7.2. Determination of the Reducing Activity of DPPH Radical

To a methanolic solution of DPPH (0.1 mM) an equal volume solution of the compounds (0.1 mM) in methanol was added. Absolute methanol was also used as control solution. The absorbance at 517 nm was recorded at room temperature after 30 and 60 min, in order to examine the possible existence of a potential time-dependence of the DPPH radical scavenging activity [64]. The DPPH scavenging activity of the compounds was expressed as the percentage reduction of the absorbance values of the initial DPPH solution (DPPH%). NDGA and BHT were used as reference compounds. 

#### 4.7.3. Assay of Radical Cation ABTS Scavenging Activity

Initially, a water solution of ABTS was prepared (2 mM). ABTS radical cation (ABTS^+●^) was produced by the reaction of ABTS stock solution with potassium persulfate (0.17 mM) and the mixture was stored in the dark at room temperature for 12–16 h before its use. The ABTS was oxidized incompletely because the stoichiometric reaction ratio of ABTS and potassium persulfate is 1:0.5. The absorbance became maximal and stable only after >6 h of reaction although the oxidation of the ABTS started immediately. The radical was stable in this form for >2 days when allowed to stand in the dark at room temperature. Afterwards, the ABTS^+●^solution was diluted in ethanol to an absorbance of 0.70 at 734 nm and 200 μL of diluted compounds or standards (0.1 mM) in DMSO were added. The absorbance was recorded out exactly 15 min after initial mixing [64]. The radical scavenging activity of the compounds was expressed as the percentage inhibition of the absorbance of the initial ABTS solution. Trolox was used as an appropriate standard.

### 4.8. CT DNA Interaction 

In vitro interaction of **3** and **4** with calf-thymus (CT) DNA was evaluated by UV-visible spectroscopy. The potential binding mode of the compounds and their corresponding binding constants (K_b_) were also determined. Additional control experiments with 5% DMSO (*v*/*v*) did not lead to any changes in the spectra of CT DNA. The UV-visible spectra of CT DNA solution (1.5 × 10^−4^ M) were recorded in the presence of each compound at diverse [compound]/[DNA] mixing ratios (r). In parallel, the UV-visible spectra of the compounds were recorded for a standard concentration (10–50 μM) in the presence of increasing amounts of CT DNA at diverse [DNA]/[compound] ratios r΄. The DNA-binding constant (K_b_, in M^−1^) was obtained by monitoring the changes in the absorbance at the corresponding λ_max_ with increasing concentrations of CT DNA and it is given by the ratio of slope to the y intercept in plots [DNA]/(ε_A_-ε_f_) versus [DNA], according to the Wolfe–Shimer equation (Equation (1)):

(1) $$\frac{\left[\mathrm{DNA}\right]}{{(\varepsilon }_{A}-\varepsilon_{f})}=\frac{\left[\mathrm{DNA}\right]}{{(\varepsilon }_{b}-\varepsilon_{f})}+\frac{1}{K_{b}{(\varepsilon }_{b}-\varepsilon_{f})}$$

where [DNA] is the concentration of DNA in base pairs, ε_A_ = A_obsd_/[compound], ε_f_ = the extinction coefficient for the free compound and ε_b_ = the extinction coefficient for the compound in the fully bound form.

The viscosity of DNA ([DNA] = 0.1 mM) in buffer solution (150 mM NaCl and 15 mM trisodium citrate at pH 7.0) was measured in the presence of increasing amounts of **3** and **4** (up to the r value = 0.35). All measurements were performed at room temperature. The obtained data are presented as (η/η_0_)^1/3^ versus r where η is the viscosity of DNA in the presence of the studied compound, η_0_ is the viscosity of DNA alone in buffer solution and r = [complex]/[DNA]. DNA-length (L/L_0_), according to the equation L/L_0_ = (η/η_0_)^1/3^. In general, the observed changes of the relative DNA-viscosity occurring in the presence of increasing amounts of the compounds which interact with CT DNA may reveal subsequently the possible binding mode. In the particular case of intercalation, an increase in the separation distance of the DNA-base pairs can be induced in order to host the inserting compound resulting in an increase of the relative DNA-viscosity. On the other hand, when a compound binds to DNA-grooves via non-classic intercalation (i.e., electrostatic interaction or external groove-binding) the DNA-viscosity may show a slight decrease or remain unchanged, because of a bend of DNA double helix which leads to shortening of DNA length [65].

Fluorescence emission spectroscopy was carried out to determine the ability of **3** and **4** to compete EB for the DNA-intercalating sites and displacing it from the EB–DNA conjugate [35]. The EB–DNA complex was prepared by adding 20 μM EB and 26 μM CT DNA in buffer solution (150 mM NaCl and 15 mM trisodium citrate at pH 7.0). The potential EB-displacing ability of the compounds was studied by a stepwise addition of a compound’s solution into the EB–DNA solution. The resultant changes were monitored by recording the variation of fluorescence emission spectra with excitation wavelength at 540 nm. The compounds did not exhibit any appreciable fluorescence emission bands at room temperature in solution or in the presence of DNA or EB under the same experimental conditions (λ_exc_ = 540 nm); therefore, the observed quenching may be attributed to the displacement of EB from its EB–DNA conjugate. The Stern-Volmer constant (K_SV_, in M^−1^) is used to evaluate the quenching efficiency for each compound according to the Stern-Volmer equation (Equation (2)) [66,67]:

(2) $$\frac{Io}{I}=1+k_{q}\tau_{0}[Q]=1+K_{SV}[Q]$$

where I_o_ and I are the emission intensities of the EB–DNA solution in the absence and the presence of the quencher, respectively, [Q] is the concentration of the quencher, τ_o_ = the average lifetime of the emitting system without the quencher and k_q_ = the quenching constant. K_SV_ may be obtained from the Stern-Volmer plots by the slope of the diagram Io/I versus [Q]. Taking τ_o_ = 23 ns as the fluorescence lifetime of the EB–DNA system, the quenching constants (k_q_, in M^−1^s^−1^) of the compounds can be determined according to Equation (3):

K_SV_ = k_q_τ_o_ (3)

### 4.9. Serum Albumins Binding 

The serum albumin (SA) binding study was performed by tryptophan fluorescence quenching experiments using BSA (3 μM) or HSA (3 μM) in buffer (containing 1 mM trisodium citrate and 150 mM NaCl at pH 7.0). The quenching of the emission intensity of tryptophan residues of BSA or HSA was monitored using compounds **3** and **4** as quenchers at increasing concentrations. The fluorescence emission spectra were recorded from 300 to 500 nm at excitation wavelength of 295 nm. The solutions of the compounds exhibited a very-low-intensity emission band at 330 nm, upon excitation at 295 nm. The fluorescence emission spectra of **3** and **4** were subtracted from the SA-emission spectra in order to proceed to calculating of the corresponding constants. 

In the meanwhile, the influence of the inner-filter effect on the measurements was negligible as evaluated by the equation:

(4) $$I_{corr}=I_{meas}\times 10^{\frac{\varepsilon_{\lambda \left(exc\right)}cd}{2}}\times 10^{\frac{\varepsilon_{\lambda \left(em\right)}cd}{2}}\;$$

where *I_corr_* = corrected intensity, *I_meas_* = the measured intensity, *c* = the concentration of the quencher, *d* = the cuvette (1 cm), *ε_λ(exc)_* and *ε_λ(em)_* = the *ε* of the quencher at the excitation and the emission wavelength, respectively, as calculated from the UV-visible spectra of the compounds. 

The Stern-Volmer and Scatchard graphs are used in order to study the interaction of a quencher with serum albumins. According to Stern-Volmer quenching equation (Equation (2)), where I_o_ = the initial tryptophan fluorescence intensity of SA, I = the tryptophan fluorescence intensity of SA after the addition of the quencher, k_q_ = the quenching constant, K_SV_ = the Stern-Volmer constant, τ_o_ = the average lifetime of SA without the quencher, [Q] = the concentration of the quencher. K_SV_ (M^–1^) can be obtained by the slope of the diagram I_o_/I versus [Q], and subsequently the quenching constant (k_q_, M^–1^s^–1^) is calculated from Equation (3) with τ_0_ = 10^–8^ s as fluorescence lifetime of tryptophan in SA. 

From the Scatchard equation

(5) ΔΙΙοQ=nK−kΔΙΙο

where, *n* is the number of binding sites per albumin and *K* is the SA-binding constant, *K* (in M^–1^) is calculated from the slope in plots ΔΙΙοQ versus $\frac{\Delta Ι}{Ιο}$ and n is given by the ratio of y intercept to the slope [21].

### 4.10. Molecular Docking Calculations

The in silico predictive tools that were employed to study the interaction of the studied compound with the selected macromolecule, are Schrödinger, Mercury, Spartan’14, and PyMOL molecular modeling software. The atomic coordinates of the compound were generated from its X-ray crystal structure as CIF file. Mercury software (http://www.ccdc.cam.ac.uk/) was used to convert the CIF file to PDB format file. The best, most stable (lowest energy) conformation of the molecular model of the compound was detected by geometrical optimization in the gas phase, as implemented in the Spartan’14 Molecular Modeling program suite [68]. The structure was initially optimized (via energy minimization) by conformational search using the Monte Carlo method with the MMFF94 molecular mechanics model, included in the Spartan’14 program suite. Geometry optimization (leading to the most stable conformer with the lowest energy) was accomplished via quantum-chemical calculations by utilizing Density functional theory (DFT) computations at B3LYP level of theory with 6-31G*(d,p) basis set to describe the accurate structural and electronic properties of the compounds, implemented by Spartan’14 program suite. Molecular docking was carried out on the crystal structure of fibroblast growth factor receptor (FGFR1) (PDB entry code 4V04), to investigate the effect of the compound on this target. X-ray crystal structure of FGFR1 in complex with its inhibitor drug ponatinib [54] was obtained from the Brookhaven Protein Data Bank (operated by the Research Collaboratory for Structural Bioinformatics, RCSB) [69,70,71]. The crystal structure of FGFR1 bound with ponatinib has been refined at 2.12 Å resolution. For the docking calculations on the kinase domain of FGFR1 it was used only the A chain of the protein since chain B is replicate, with ponatinib (0LI) FGFR1 inhibitor bound at the same ligand binding site among the chains. For this reason, from the corresponding PDB files, the data for chain B and the data of the drugs referring to this chain were deleted. In our studies, molecular docking calculations were performed with Schrödinger modeling suite having the ability for accurate calculations. The Schrödinger software suite contains a broad array of computational chemistry tools. In the procedure for molecular docking with the employment of Schrödinger suite all compounds were sketched and converted into three-dimensional MOL2 files using Schrödinger Release 2015-2 Maestro Version 10.5 and minimized using LigPrep 3.4 [72] (which can generate a number of structures from each input structure with various ionization states, tautomers, stereochemical characteristics, and ring conformations to eliminate molecules on the basis of various criteria such as molecular weight or specified numbers and types of functional groups with correct chiralities for each successfully processed input structure), and the OPLS3 (**O**ptimized **P**otential for **L**iquid **S**imulations) [73] force field for the optimization, producing the low-energy isomers of the ligands (Schrödinger, http://www.schrodinger.com). Energy minimized 3D molecular structures were generated with the employment of LigPrep run from Maestro utility of the Schrödinger suite. The ligand preparation included 2D–3D conversions, generating variations, correction, verification and optimization of the structures. A preparation of receptor and ligand structures was integrated before the actual docking procedure [74]. The crystal structure of the protein was prepared using the Protein Preparation Wizard, in Schrödinger Suite 2018-2 (Schrödinger, LLC, New York, NY) [75,76]. Protein was prepared by adding the hydrogen atoms, optimizing hydrogen bonds, removing atomic clashes, adding formal charges to the hetero groups and then optimizing at neutral pH. Missing loops and side chains were prepared using Prime version 3.2 [77,78]. Finally, the structure was minimized using OPLS3 force field. Active site of studied proteins was obtained using SiteMap tool [79,80], which provides a fast and effective means of identifying potential binding pockets of proteins. SiteMap identifies the character of binding sites using novel search and assesses each site by calculating various properties like size, volume, amino acid exposure, enclosure, contact, hydrophobicity, hydrophilicity and donor/acceptor ratio. Receptor grid was generated around the active site for effective binding using Receptor grid generation in the Glide (version 5.9) application of Maestro. Once the receptor grid is generated, the ligand is docked to the protein using Glide docking tool of Schrödinger (**G**rid based Ligand **D**ocking with **E**nergetics) [80,81]. The studied compound was docked in the binding site of the protein using Induced-Fit Docking (IFD) protocol 2015-2 [82,83] The ligand interactions are shown in Ligand interaction tool of Maestro (Schrödinger). Waters were deleted with Maestro, the graphical user interface (GUI) of Schrödinger software, prior to docking. Molecular docking studies were carried out for the best fitted compounds to the model, while the final selection criteria were compounds docking scores and the presence of crucial interactions for binding to the studied proteins [84]. The resulting poses were examined manually and the most promising ones were redocked with IFD calculations. Poses that pass the initial screens enter the final stage of the algorithm, which involves evaluation and minimization of a grid approximation to the OPLS-AA nonbonded ligand-receptor interaction energy. Final scoring is then carried out on the energy-minimized poses. By default, Schrödinger’s proprietary GlideScore [81] multi-ligand scoring function is used to score the poses. The compound showed good docking scores reflecting drug-binding affinities with the studied proteins. The PyMol Molecular Graphics System (Schrödinger, LLC. version 1.8.2.0, www.pymol.org), was used to visualize the molecules and analyze the results of the docking and to construct the molecular models [85].

## Figures and Tables

**Table 1:** Selected bond lengths (Å) and angles (°) for **3** and **4**.

	3	4
Bond lengths (Å)		
Ag1–P1	2.513(1)	2.451(1)
Ag1–P2	2.471(1)	2.449(1)
Ag1–S1	2.699(2)	2.654(1)
Ag1–Cl1	2.561(1)	
Ag1···O1		2.768(3)
Bond angles (°)		
P1–Ag1–P2	109.68(3)	130.69(3)
P2–Ag1–S1	103.31(3)	112.24(3)
P1–Ag1–S1	108.38(4)	102.89(3)
Ag1–S1–C1	99.98(14)	111.7(2)
P1–Ag–Cl1	113.00(3)	
P2–Ag–Cl1	121.74(3)	
S1–Ag–Cl1	98.88(3)	

**Table 2:** Photophysical characteristics of compounds **1**–**4**: absorption band maxima λ_max_(abs) (in nm) and molar absorption coefficients ε (M^−1^cm^−1^) in CH_2_Cl_2_ solutions (~5 × 10^−5^ M), and emission band maxima λ_max_(em) (in nm) in DMSO solutions (~5 × 10^−5^ M).

Compound	λ_max_(abs)/nm (ε/M^−1^cm^−1^)	λ_max_(em)/nm
**1**	256 (26,500), 310 (3300)	428
**2**	257 (37,000)	460
**3**	254 (16,300), 307 (10,300)	432
**4**	265 (30,700)	400, 419, 434

**Table 3:** Spectral changes in UV absorption bands of compounds **3**, **4** and mtdztH in DMSO solutions (~2.5–5 × 10^‒5^ M) upon addition of increasing concentrations of CT DNA: wavelength of UV absorption band maximum (λ_max_, in nm), percentage of hyper-/hypochromism (ΔA/A⁠_o_, in %), blue/red shift of λ⁠_max_ (Δλ, in nm) and CT DNA binding constants (K⁠_b_, in M⁠^−1^).

Compound	λ_max_(nm) (ΔA/A_ο_ (%) ^a^, Δλ (nm) ^b^)	K_b_ (M^−1^)
**3**	275 nm (+15, +2)	2.48 (±0.32) × 10^4^
**4**	272 nm (–13, 0 )	6.63 (±0.12) × 10^5^
mtdztH	313 (–12,0)	7.34 (±0.02) × 10^7^

a “+” denotes hyperchromism, “‒” denotes hypochromism; b “+” denotes red shift, “‒” denotes blue shift.

**Table 4:** Percentage of EB–DNA fluorescence quenching (ΔI/Io, in %), EB-DNA Stern–Volmer constants (K_SV_ in M^−1^) and EB–DNA quenching constants (k_q_, M^−1^s^−1^) for compounds **3** and **4**.

Compound	ΔI/I_ο_ (%)	K_SV_ (M^−1^)	k_q_ (M^−1^s^−1^)
**3**	25.3	2.40 (±0.08) × 10^4^	1.40 (±0.03) × 10^12^
**4**	27.6	5.68 (±0.20) × 10^4^	2.47 (±0.10) × 10^12^

**Table 5:** Emission-quenching constants (k_q_) and binding constants (K) for the interaction of **3** and **4** with BSA and HSA.

Compound	BSA		HSA	
	k_q_ (M^−1^s^−1^)	K (M^−1^)	k_q_ (M^−1^s^−1^)	K (M^−1^)
**3**	6.62 (±0.29) × 10^12^	7.89 (±0.8) × 10^4^	5.91 (±0.25) × 10^12^	1.45 (±0.11) × 10^5^
**4**	7.15 (±0.40) × 10^12^	1.29 (±0.09) × 10^5^	5.11 (±0.37) × 10^11^	7.21 (±0.53) × 10^4^

## Data Availability

Not applicable.

## Supplementary Materials

The following supporting information can be downloaded at: https://www.mdpi.com/article/10.3390/molecules28010336/s1. Sections: S1.1. Single-crystal X-ray structural analysis (Figures S1–S3 and Tables S1–S3: X-ray crystal structures of **3** and **4**); S1.2. Stability in solution (Figure S4: Stability test of **1**–**4** by UV–visible spectroscopy); S1.3. In vitro antibacterial activity (Table S4: MIC and IC_50_ values of the in vitro antibacterial activity of **1**–**4** and their corresponding ligands); S1.4. In vitro anti-cancer activity (Table S5: GI_50_, TGI, and IC_50_ values of the in vitro anti-cancer activity of **1**–**4**); S1.5. In vitro antioxidant activity (Table S6: In vitro antioxidant activity of **1**–**4** and their corresponding ligands); S1.6. CT DNA interaction (Figures S5–S7: Spectrophotometric studies of the interaction of compounds with CT DNA); S1.7. Serum albumin binding (Figures S8–S11: Spectrophotometric studies of the interaction of compounds with HSA and BSA). Accession Codes: CCDC 2179510 and 2179511 contain the supplementary crystallographic data for compounds **3** and **4**, respectively. These data can be obtained free-of-charge via http://www.ccdc.cam.ac.uk/conts/retrieving.html, or from the Cambridge Crystallographic Data Centre, 12 Union Road, Cambridge CB2 1EZ, UK; Fax: (+44) 1223-336-033; or email: deposit@ccdc.cam.ac.uk.

## Sample Availability

Samples of the compounds are available from the authors upon request.
