# Supplementary material for: Inhibition of Cancer Cell Proliferation and Bacterial Growth by Silver(I) Complexes Bearing a CH3-Substituted Thiadiazole-Based Thioamide
Source: Molecules. 2023 Jan 1;28(1):336. doi: 10.3390/molecules28010336 (PMC9823356; doi:10.3390/molecules28010336)
Supplement: Supplementary file 1 [file molecules-28-00336-s001.zip › molecules-2102347-supplementary.pdf]

Supplementary information

Inhibition of cancer cell proliferation and bacterial growth by silver(I) complexes bearing a CH_3-substituted thiadiazole-based thioamide

Despoina Varna [1], Elena Geromichalou [2], Georgia Karlioti [1], Rigini Papi [3], Panagiotis Dalezis [2], Antonios G. Hatzidimitriou [1], George Psomas [1], Theodora Choli-Papadopoulou [3], Dimitrios T. Trafalis [2,*] and Panagiotis A. Angaridis [1,*]

[1] *Laboratory of Inorganic Chemistry, Department of Chemistry, Aristotle University of Thessaloniki, 54124 Thessaloniki, Greece*

[2] *Laboratory of Pharmacology, Medical School, National and Kapodistrian University of Athens, 75 Mikras Asias Street, 11527 Athens, Greece*

[3] *Laboratory of Biochemistry, Department of Chemistry, Aristotle University of Thessaloniki, 54124 Thessaloniki, Greece*

TABLE OF CONTENTS

S1.1 Single-crystal X-ray structure analysis 3
S1.2 Stability in solution 7
S1.3 In vitro antibacterial activity 8
S1.4 In vitro anticancer activity 9
S1.5 In vitro antioxidant activity 10
S1.6 CT DNA interaction 11
S1.6.1 CT DNA interaction followed by UV absorption spectroscopy 11
S1.6.2 EB-DNA competitive binding 13
S1.7 Serum albumins binding 14

S1.1 Single-crystal X-ray structure analysis

Table S1. Crystal data, data collection, and refinement parameters for **3** and **4.**

	**3**	**4**
Chemical formula	$C_{42}H_{36}AgClN_2OP_2S_2$	$C_{29}H_{28}AgN_3O_3P_2S_2$
Formula weight	854.16	700.51
Crystal system	Monoclinic	Monoclinic
Space group	$P2_1/c$	$P2_1/n$
Temperature (K)	295	295
Unit cell parameters		
a (Å)	21.6391(15)	14.5156(15)
b (Å)	9.4529(7)	9.3682(10)
c (Å)	19.4262(14)	22.602(2)
α (°)	90	90
β (°)	99.480(2)	92.677(3)
γ (°)	90	90
Volume ($Å^3$)	3919.4(5)	3070.2(5)
Z	4	4
Radiation type, *λ* (Å)	Mo *Kα*	Mo *K*α
Absorption coefficient (mm^{-1})	0.81	0.93
Crystal size (mm)	0.26×0.22×0.16	0.12×0.11×0.09
Diffractometer	Bruker Kappa Apex2	Bruker Kappa Apex2
Absorption correction	Numerical<br>Analytical Absorption (De Meulenaer & Tompa, 1965)	Numerical<br>Analytical Absorption (De Meulenaer & Tompa, 1965)
T_{min}, T_{max}	0.84, 0.88	0.90, 0.92
Number of measured, independent and observed [I> 2.0σ(I)] reflections	44566, 7417, 5256	37287, 5849, 4230
R_{int}	0.030	0.042
$(\sin \theta/\lambda)_{max}$ ($Å^{-1}$)	0.611	0.613
$R[F^2>2\sigma(F^2)]$, $wR(F^2)$, S	0.038, 0.060, 1.00	0.040, 0.052, 1.00
No. of reflections	5256	4230
No. of parameters	460	361
$\Delta\rho_{max}$, $\Delta\rho_{min}$ (e $Å^{-3}$)	0.53, −0.72	0.62, − 0.51

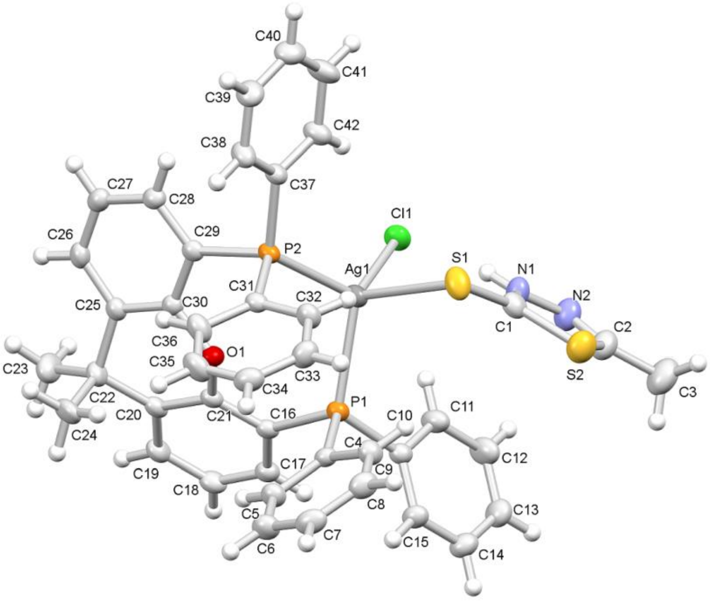

Figure S1. Crystal structure of [AgCl(mtdztH)(xantphos)] (**3**). Atoms are presented as thermal ellipsoids at the 25% probability level as well as Hydrogen atoms are shown as spheres of arbitrary radius.

Table S2. Selected bond lengths (Å) and angles (°) for **3**.

Bond lengths (Å)			
Ag1–P1	2.513(1)	Ag1–S1	2.699(2)
Ag1–P2	2.471(1)	Ag1–Cl1	2.561(1)
Bond angles (°)			
P1–Ag1–P2	109.68(3)	P1-Ag1-Cl1	113.00(3)
P1–Ag1–S1	108.38(4)	P2-Ag1-Cl1	121.74(3)
P2–Ag1–S1	103.31(3)	Cl1–Ag–S1	98.88(3)
Ag1–S1–C1	99.98(14)		

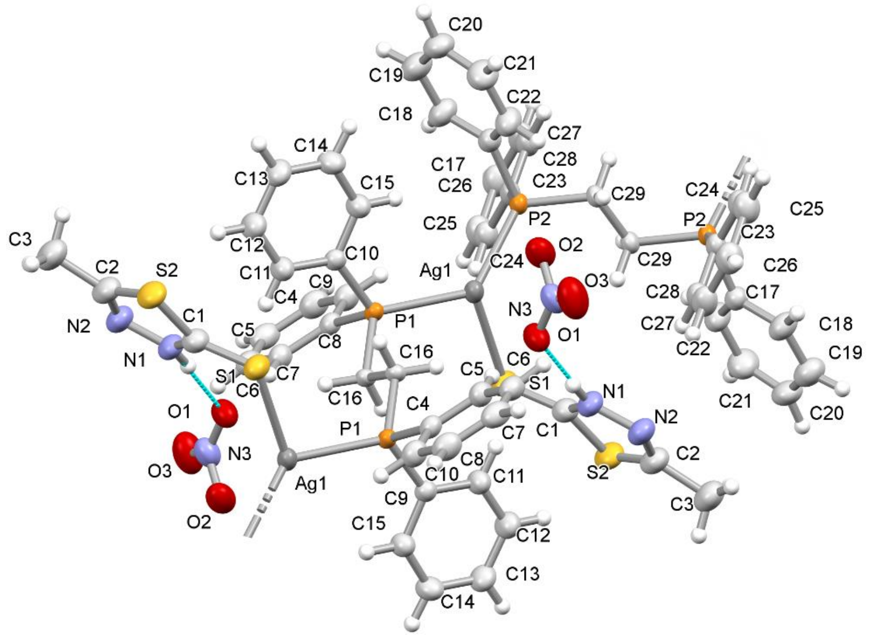

Figure S2. View of the crystal structure of part of the polymeric chain of $[Ag(mtdztH)(dppe)(NO_3)]_n$ (**4**). Atoms are presented as thermal ellipsoids at the 25% probability level as well as Hydrogen atoms are shown as spheres of arbitrary radius.

Table S3. Selected bond lengths (Å) and angles (°) for **4**.

Bond lengths (Å)			
Ag1–P1	2.451(1)	Ag1–S1	2.654(1)
Ag1–P2	2.449(1)	Ag1···O1	2.768(3)
**Bond angles (°)**			
P1–Ag1–P2	130.69(3)	P2–Ag1–S1	102.89(3)
P1–Ag1–S1	112.24(3)	Ag1–S1–C1	111.7(2)

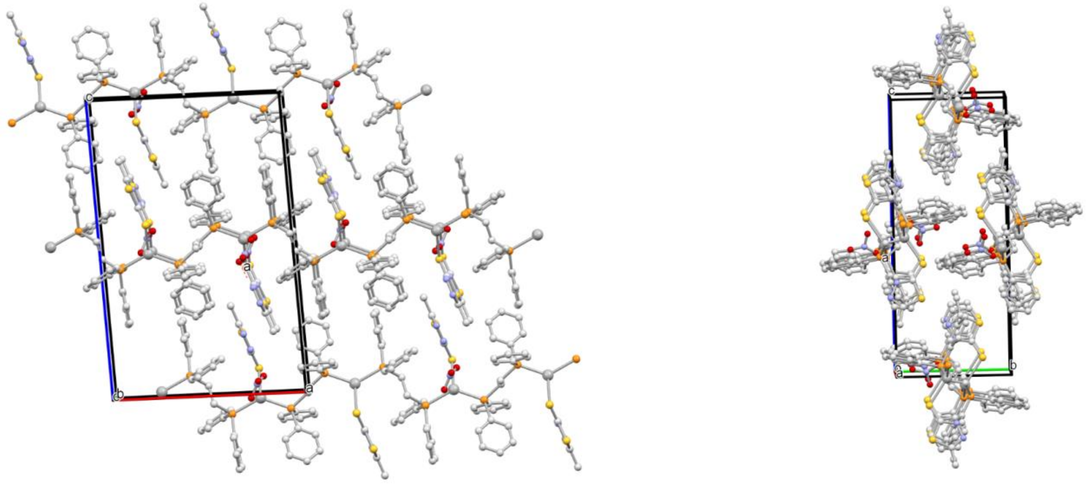

Figure S3. Packing diagrams for **4** showing its parallel one-dimensional zigzag chains along the a-axis.

S1.2 Stability in solution

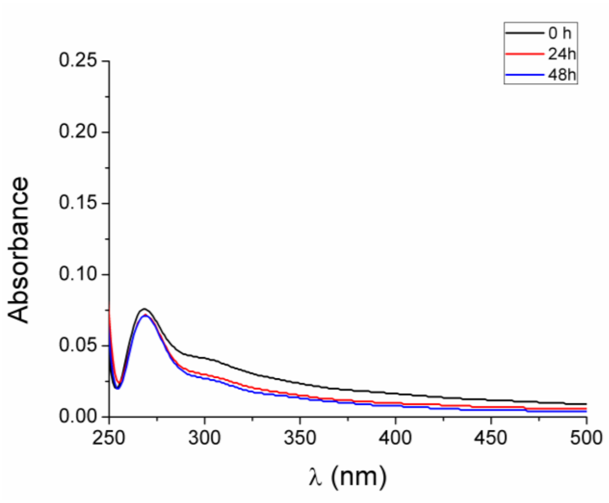

$[AgCl(mtdztH)(PPh_3)_2]$ (**1**)

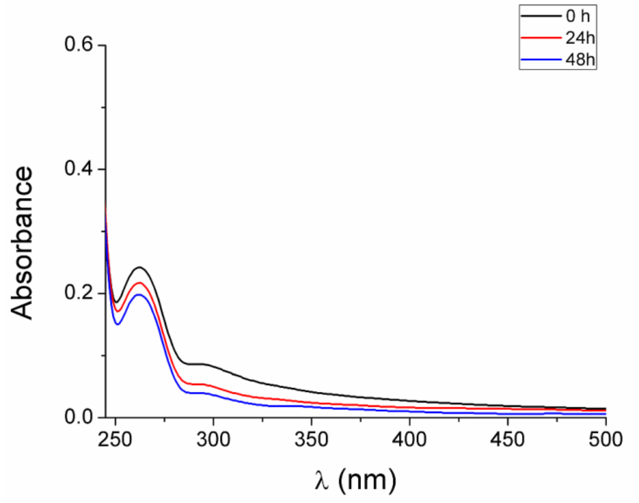

$[Ag(mtdzt)(PPh_3)_3]$ (**2**)

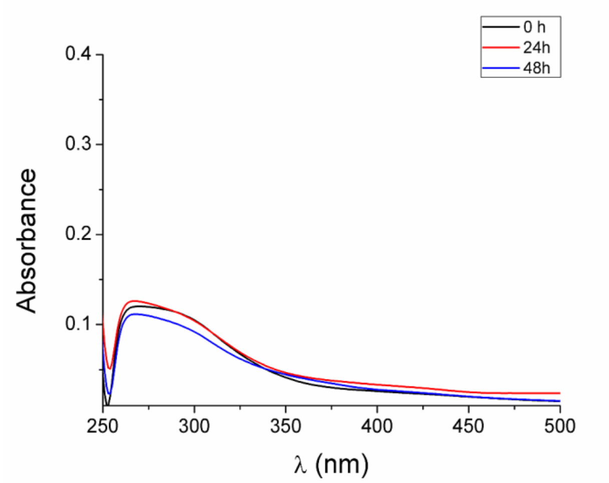

[AgCl(mtdztH)(xantphos)] (**3**)

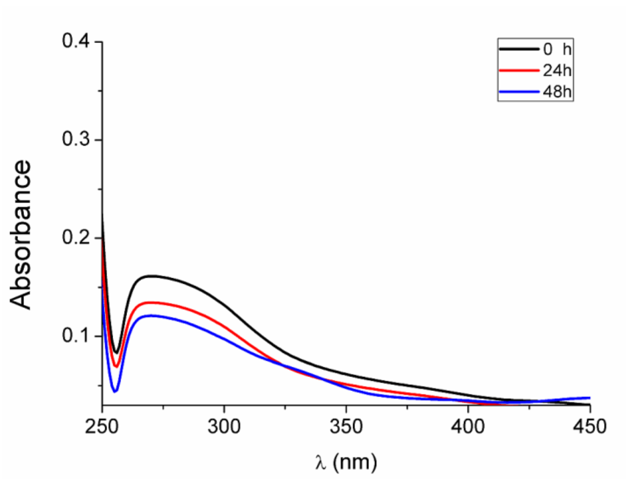

$[Ag(mtdzt)(dppe)(NO_3)]_n$ (**4**)

Figure S4. UV-visible absorption spectra of **1-4** in PBS saline buffer solutions (10^{-6} M, pH = 7.4) in 0, 24 and 48 h time intervals.

S1.3 In vitro antibacterial activity

Table S4. In vitro antibacterial activity of **1-4**, and their respective ligands mtdztH, xantphos, PPh_3, and dppe in free form, evaluated by the minimum inhibitory concentration (MIC) and the half-minimum inhibitory concentration (IC_{50}) values (in μg/mL, and in μM of values in parentheses) provided by a nonlinear curve fit-growth/sigmoidal-dose response on the experimental optical density data. Values are expressed as mean ± standard deviation (SD) of three replicate measurements (with the exception of values higher than 100 μg/mL). Ampicillin was used as a reference showing MIC = 100 μg/mL against *E.coli* (BL21) bacterial cell line.

Compounds	***E. coli***		***S. aureus***		***B. subtilis***		***B. cereus***	
	**MIC**	**IC_{50}**	**MIC**	**IC_{50}**	**MIC**	**IC_{50}**	**MIC**	**IC_{50}**
	**μg/mL (μM)**		**μg/mL (μM)**		**μg/mL (μM)**		**μg/mL (μM)**	
$[AgCl(mtdztH)(PPh_3)_2]$ (**1**)	>100	>100	100	16 (20±3.2)	100	21 (26±4.0)	100	81 (101±10.3)
$[Ag(mtdzt)(PPh_3)_3]$ (**2**)	>100	>100	100	17 (16±2.6)	100	20 (19±2.8)	100	12.5 (12±1.4)
[AgCl(mtdztH)(xantphos)] (**3**)	80 (94)	8.1 (9.5±0.1)	100 (117)	6.1 (7.1±1.04)	72 (84)	12 (14±0.6)	54 (65.5)	26 (30±2.1)
$[Ag(dppe)(mtdztH)(NO_3)]_n$ (**4**)	>100 (91)	11 (15.4±2.4)	50 (45)	8 (7.3±1.6)	30 (27)	4.9 (4.5±0.8)	50 (45)	5 (4.6±0.7)
mtdztH	>100	30 (227±6.0)	>100	21 (159±9.0)	>100	53 (401± 16.2)	>100	21 (159±8.9)
dppe	>100	>100	>100	>100	>100	>100	>100	>100
xantphos	>100	50 (86±6.9)	>100	78 (135±7.5)	>100	65 (112±6.5)	>100	65 (112±6.9)
PPh_3	>100	51 (194±2.0)	>100	70 (267±2.2)	>100	>100 (>380)	>100	70 (267±9.0)

S1.4 In vitro anticancer activity

Table S5. In vitro growth inhibition/cytostatic (GI_{50} and TGI, in μM) and cytocidal/cytotoxic (IC_{50}, in μM) effects induced by **1-4** against SKOV-3, Hup-T3, DMS114, and PC3 human cancer, and MRC5 human normal cell lines. Cisplatin was used as a reference showing GI_{50} = 8±1, TGI = 24±2 and IC_{50} = 36±2 against MRC5 human normal cells.

Compound	MRC5			SKOV-3			Hup-T3			DMS114			PC3		
	GI_{50}	TGI	IC_{50}	GI_{50}	TGI	IC_{50}	GI_{50}	TGI	IC_{50}	GI_{50}	TGI	IC_{50}	GI_{50}	TGI	IC_{50}
	(μM)			(μM)			(μM)			(μM)			(μM)		
**1**	3.0±0.5	5.2±0.5	9.2±0.8	1.2±0.2	3.1±0.4	5.7±0.4	2.2±0.2	4.1±0.3	9.2±0.3	1.9±0.1	3.1±0.1	4.5±0.3	3.0±0.3	5.7±0.5	9.6±0.5
**2**	2.0±0.02	3.0±0.05	4.1±0.06	1.9±0.2	3.0±0.3	4.1 ±0.3	1.9±0.2	3.2±0.2	4.5±0.2	1.2±0.05	2.6±0.1	4.0±0.25	2.0±0.05	3.2±0.12	4.4±0.15
**3**	14.0±1.2	19.0±1.5	24.0±1.8	8.0±0.5	10.0±0.5	16.0 ±0.8	7.0±0.4	12.0±0.6	24.0±0.6	5.0±0.2	8.0±0.2	11.0±0.4	7.0±0.7	9.5±0.8	12.0±0.9
**4**	1.2±0.05	2.8±0.05	4.4±0.08	1.0±0.02	2.0±0.03	2.3 ±0.05	0.4±0.02	0.9±0.04	3.0±0.04	0.5±0.04	1.0±0.05	3.1±0.05	0.6±0.03	1.6±0.03	3.6±0.05

S1.5 In vitro antioxidant activity

Table S6. In vitro H_2O_2 reducing ability (%H_2O_2) as well as DPPH (%DPPH) and ABTS (%ABTS) scavenging activity of **1**-**4**, mtdztH, PPh_3, xantphos, dppe and the reference compounds NDGA, BHT, Trolox, L-ascorbic acid.

**Compound**	**%H_2O_2**	**%DPPH**		**%ABTS**
		**30 min**	**60 min**	
[AgCl(mtdztH)(PPh_3)$_2$] (**1**)	74.85±0.79	44.00±1.13	46.31±0.81	71.55±1.31
[Ag(mtdzt)(PPh_3)$_3$] (**2**)	80.59±0.55	54.36±1.28	61.84±0.94	90.73±0.39
[AgCl(mtdzt)(xantphos)] (**3**)	60.47±0.79	17.36±0.57	19.09±0.47	91.27±1.63
[Ag(mtdzt)(dppe)(NO_3)]$_n$ (**4**)	35.17±1.96	34.39±0.29	40.55±2.23	65.03±0.26
mtdztH	97.17±0.10	32.81±1.18	52.55±1.31	75.97±0.65
xantphos	98.18±0.75	22.01±0.13	33.23±0.27	51.11±1.06
PPh_3	87.96±1.32	3±0.12	8.04±0.22	58.29±1.2
dppe	92.64±0.71	34.28±2.01	40.76±1.38	60.9±1.40
NDGA	Not tested	87.08±0.12	87.47±0.12	Not tested
BHT	Not tested	61.30±1.16	76.78±1.12	Not tested
Trolox	Not tested	Not tested		98.10±0.48
L-ascorbic acid	60.80±0.20	Not tested		Not tested

S1.6 CT DNA interaction

S1.6.1 CT DNA interaction followed by UV absorption spectroscopy

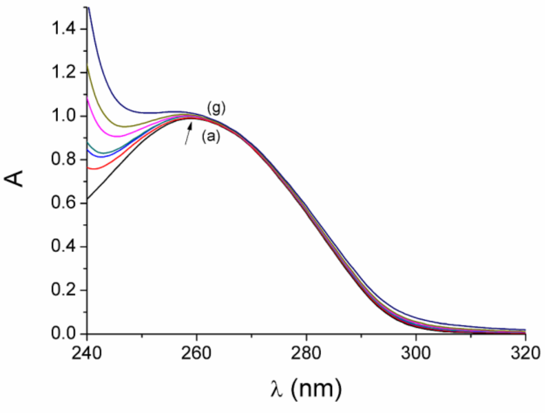

(a)

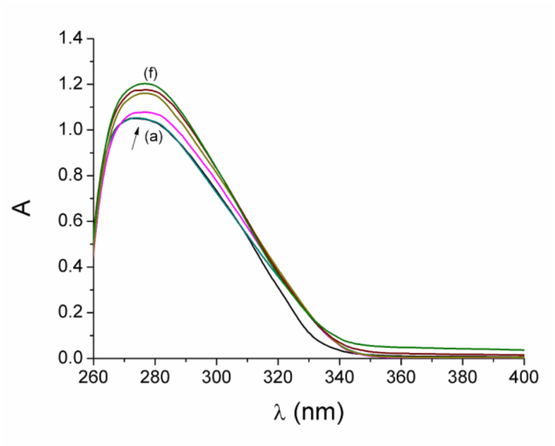

(b)

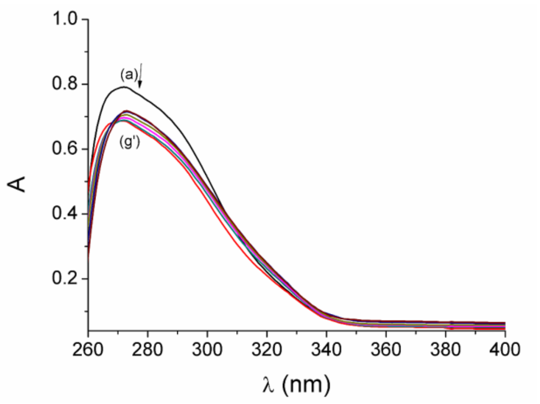

(c)

Figure S5. (a) UV absorption spectra of CT DNA (0.15 mM) in buffer solution (150 mM NaCl and 15 mM trisodium citrate at pH 7.0) in the absence and presence of increasing amounts of **3**. The arrows show the changes upon increasing concentrations of the compound (a→g). (b) and (c) UV absorption spectra of **3** and **4** in DMSO solutions (5×10^{-5} M), respectively, in the presence of increasing amounts of CT DNA (a→f for **3** and a→g' for **4**) (r' = [DNA]/[compound] = 0-0.73). The arrows show the changes upon increasing amounts of CT DNA.

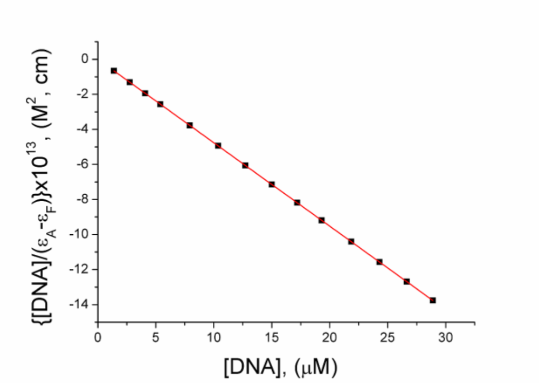

[AgCl(mtdztH)(xantphos] (**3**)

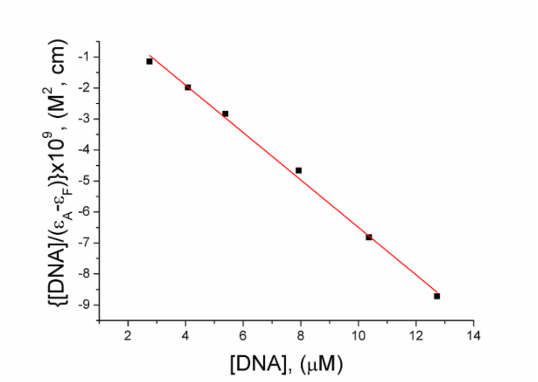

[Ag(mtdztH)(dppe)(NO_3)]$_n$ (**4**)

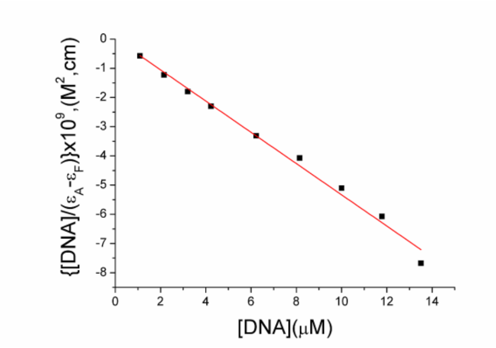

mtdztH

Figure S6. Plots of [DNA]/(εA-εf) *versus* [DNA] for **3**, **4** and mtdztH.

S1.6.2 EB-DNA competitive binding

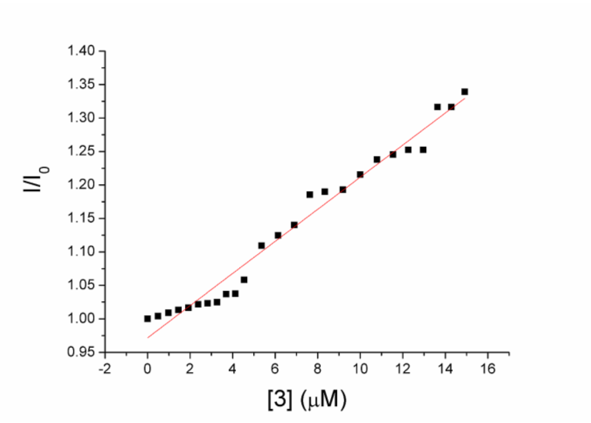

[AgCl(mtdtH)(xantphos)] (**3**)

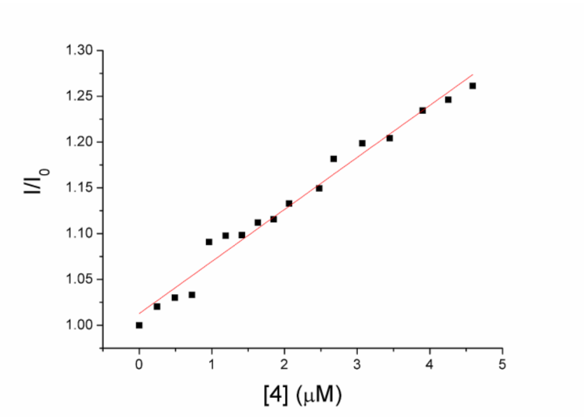

$[Ag(mtdztH)(dppe)(NO_3)]_n$ (**4**)

Figure S7. Stern-Volmer plots of quenching EB–DNA fluorescence for **3** and **4**.

S1.7 Serum albumins binding

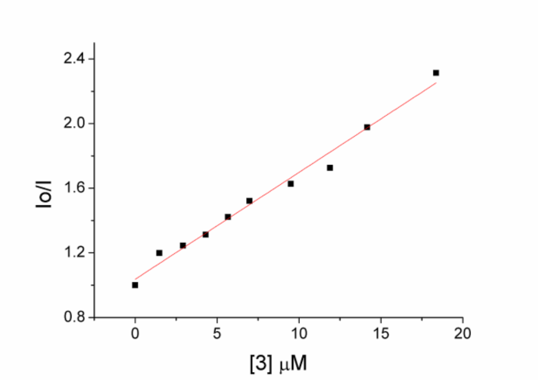

[AgCl(mtdztH)(xantphos)] (**3**)

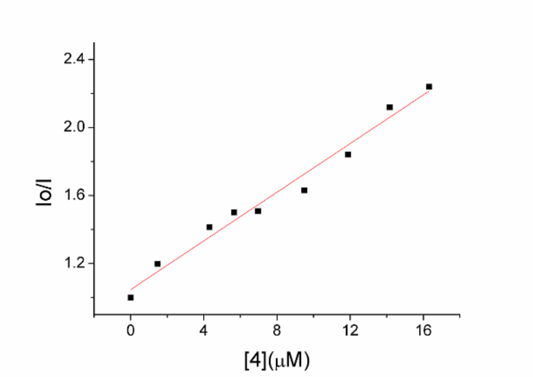

[Ag(mtdztH)(dppe)(NO_3)]$_n$ (**4**)

Figure S8. Stern-Volmer quenching plots of BSA for **3** and **4**.

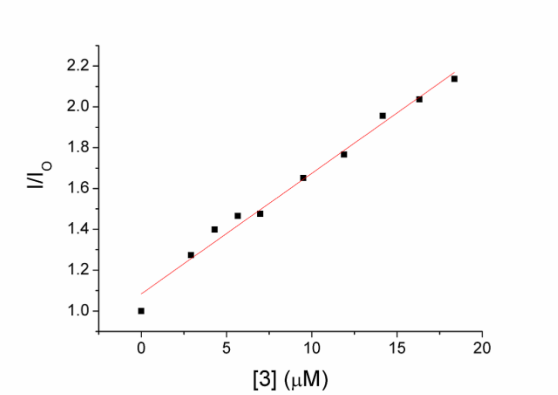

[AgCl(mtdztH)(xantphos)] (**3**)

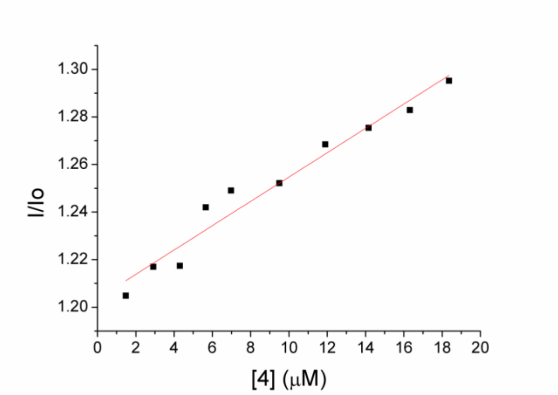

[Ag(mtdztH)(dppe)(NO_3)]$_n$ (**4**)

Figure S9. Stern-Volmer quenching plots of HSA for **3** and **4**.

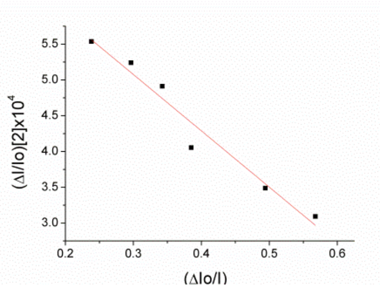

[AgCl(mtdztH)(xantphos)] (**3**)

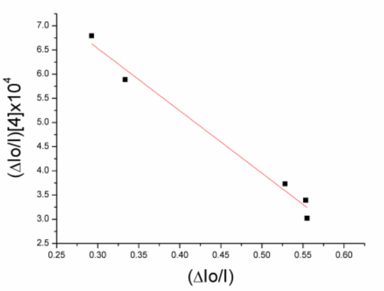

[Ag(mtdztH)(dppe)(NO_3)]$_n$ (**4**)

Figure S10. Scatchard quenching plots of BSA for **3** and **4**.

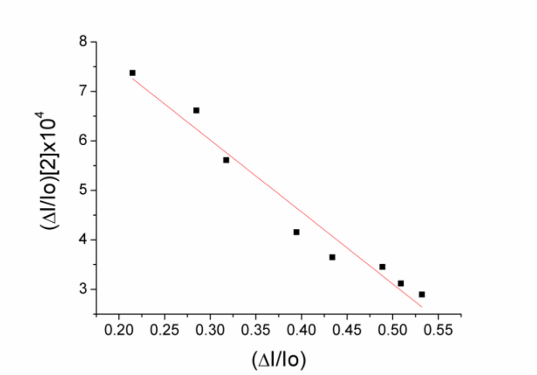

[AgCl(mtdztH)(xantphos)] (**3**)

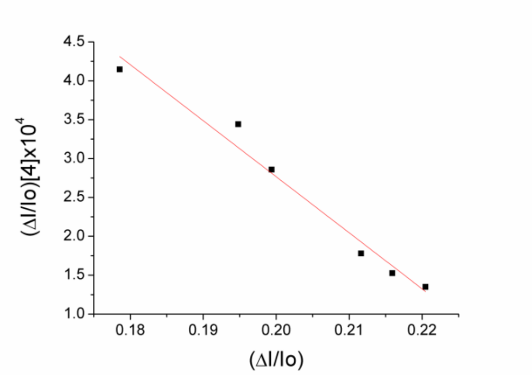

[Ag(mtdztH)(dppe)(NO_3)]$_n$ (**4**)

Figure S11. Scatchard quenching plots of HSA for **3** and **4**.
